# Electrochemical Sensors Based on Conducting Polymers for the Aqueous Detection of Biologically Relevant Molecules

**DOI:** 10.3390/nano11010252

**Published:** 2021-01-19

**Authors:** Álvaro Terán-Alcocer, Francisco Bravo-Plascencia, Carlos Cevallos-Morillo, Alex Palma-Cando

**Affiliations:** 1 Grupo de Investigación Aplicada en Materiales y Procesos (GIAMP), School of Chemical Sciences and Engineering, Yachay Tech University, Hda. San José s/n y Proyecto Yachay, 100119 Urcuquí, Ecuador; alvaro.teran@yachaytech.edu.ec (Á.T.-A.); francisco.bravo@yachaytech.edu.ec (F.B.-P.); 2 Facultad de Ciencias Químicas, Universidad Central del Ecuador, Francisco Viteri s/n y Gato Sobral, 170129 Quito, Ecuador; cacevallosm@uce.edu.ec

**Keywords:** electrochemical sensors, conducting polymers, emergent pollutants, neurotransmitters, ascorbic acid, glucose, phenolic compounds, pharmaceuticals, nitroaromatic compounds, hydrogen peroxide

## Abstract

Electrochemical sensors appear as low-cost, rapid, easy to use, and in situ devices for determination of diverse analytes in a liquid solution. In that context, conducting polymers are much-explored sensor building materials because of their semiconductivity, structural versatility, multiple synthetic pathways, and stability in environmental conditions. In this state-of-the-art review, synthetic processes, morphological characterization, and nanostructure formation are analyzed for relevant literature about electrochemical sensors based on conducting polymers for the determination of molecules that (i) have a fundamental role in the human body function regulation, and (ii) are considered as water emergent pollutants. Special focus is put on the different types of micro- and nanostructures generated for the polymer itself or the combination with different materials in a composite, and how the rough morphology of the conducting polymers based electrochemical sensors affect their limit of detection. Polypyrroles, polyanilines, and polythiophenes appear as the most recurrent conducting polymers for the construction of electrochemical sensors. These conducting polymers are usually built starting from bifunctional precursor monomers resulting in linear and branched polymer structures; however, opportunities for sensitivity enhancement in electrochemical sensors have been recently reported by using conjugated microporous polymers synthesized from multifunctional monomers.

## 1. Introduction

Neurotransmitters are molecules responsible to transmit neurological signals and permit the intercellular communication between neuron cells [1,2]. The body concentration of these molecules affects brain work, mood, pain response, and physical performance [3]. They regulate the process of consciousness, motivation, and memorization [4]. It means that the correct balance of neurotransmitters’ concentration in the body is fundamental to maintain human health, and prevent mental disorders and diseases [2]. Uric acid (UA) appears as a particularly important biomarker because it is a final product of purine metabolism and it is easily accumulated in the human body due to its low aqueous solubility, while glucose concentration is an important marker of human health, especially for symptoms associated with diabetes. The elevated concentrations of hydrogen peroxide encountered in biological fluids are generally linked with the incidence of different oxidative stress-related pathologies such as neurological disorders (Parkinson and Alzheimer), cancer, and cardiovascular diseases. Therefore, determination and quantification of the concentration of neurotransmitters, UA, glucose, and hydrogen peroxide in human fluids are critical towards a better and fast diagnostic and treatment of different diseases and disorders [5,6]. On the other hand, environmental water pollutants have become an issue of great importance during recent decades [7]. Emerging contaminants are chemicals with potential negative health effects associated with human exposure [8]. These compounds are widely released into aquatic ecosystems from agricultural, paints, textile, plastic, pharmaceutical, petroleum, and other industries [9]. These compounds can be found in aqueous environments dispersing and persisting to a great extent [10]. Among all of these pollutants, phenolic compounds, hydrazine, nitrites, pharmaceutical, and nitroaromatic compounds are especially hazardous, because of their high toxicity, carcinogenicity, and low biodegradability [11]. Skin damage, necrosis, methemoglobinemia, drowsiness, nausea, and many other symptoms have been associated with these pollutants [12,13,14]. Many contaminants are difficult to remove with conventional wastewater treatment systems, so these facilities are another source of emerging pollutants [15]. Phenols are derived from industrial wastewater [16], used in the production of aromatic compounds such as explosives, fertilizers, paint removers, textile, plastics, and drugs [17,18]. Exposures to phenolic compounds damage the lungs, liver, kidneys, and genitourinary tract [19]. Nitrites are found in food and physiological systems [20] producing carcinogenic nitrosamines [21]. As a fertilizer, it can highly impact water sources [22]. Nitroaromatics compounds are the major components of explosives whose residues can accumulate in the environment [23]. Commonly, methods such as gas chromatography, mass spectrometry [24], and high-performance liquid chromatography [25] have been used for biomarkers and emerging pollutants determination, but they are expensive, time-consuming, and require well-skilled operators and pretreated samples [26]. Electrochemical detection has proved to overcome many of the disadvantages of commonly used techniques [27,28]. Some electrochemical sensor devices have even reached commercialization and routinely usage [29]. Nevertheless, multiple efforts are oriented to the development of new electrochemical sensors due to their potential and wide application.

Currently, a huge variety of materials has been employed for building electrochemical sensors aiming to improve their properties such as electrical conductivity, surface area, and mechanical- and chemical stability [30]. The building material selection pretends to solve some problems of the electrochemical sensors like electrode fouling and overlapping of the redox potential of the molecules presented as analyte [31]. In that context, intrinsically conducting polymers (CPs) are one of the most relevant and used materials for modification of sensors by their unique physical and chemical properties [32,33] such as adjustable architecture, adaptability, versatility, room stability, and sensitivity to surfer changes in their electrochemical activity with slight changes in its surface [34,35,36]. The electrochemical sensors have evolved through the employment of novel modifier materials such as conducting polymers and carbon nanomaterials [37,38], because the immobilization transfers the physicochemical properties of the modifier to the electrode surface, showing high surface area, excellent thermal conductivity [39], high conductivity [11], and strong mechanical strength [40,41,42]. Additionally, different types of nanoparticles can be added to the polymers forming composites [43], allowing for the combination of the properties which can improve the mechanical, optical, and electrical properties of polymer without sacrificing its processability or adding excessive weight [44,45]. CPs such as polypyrroles, polyanilines, and polythiophenes have attracted considerable attention due to their good film-forming property, electrical semiconductivity, high transparency in the visible region, and excellent thermal and environmental stability [46]. Electrode surfaces are usually modified with thin CP films by a combination of adsorptive attraction and low solubility in the working solution, using pre-formed soluble polymers or electrochemical polymerization.

Over the past five years, several electrochemical sensors based on CPs have been developed by numerous research groups with major contributions in electroanalytical and materials science aiming for the determination of a wide range of analytes. Recently, Wang et al. provided a review on recent reports on electrochemical sensors based on various nanomaterial-doped conducting polymers [47], Ibanez et al. described the most important procedures to customize CPs for electroanalytical purposes [48], Park et al. discussed the prospects of scientific and technological challenges for preparing next-generation biosensors focused on CP nanomaterials [49] and Naveen et al. analyzed the applications of conducting polymer composites in electrochemical sensors [50]. Herein, an extensive state-of-the-art review is reported focusing on CPs based electrochemical sensors for detection into liquid solutions of (i) molecules with a function regulation in the human body such as dopamine, epinephrine, serotonin, uric acid, ascorbic acid, glucose, and hydrogen peroxide, and (ii) water emergent pollutants such as phenolic compounds, hydrazine, nitrites, pharmaceuticals, and nitroaromatic compounds. Special focus is put on the formation of nanostructures during the synthesis of the CPs which usually results in mesoporous and macroporous structures with large surface areas as a recurrent approach for increasing the sensor’s sensitivity. Considering the copious and highly dispersed information in the literature, it has become challenging to include all the information reported in scientific journals, therefore we apologize for any work that was not included in this document. This review includes a short section of fundamentals in the electrochemical sensors followed by reports of the last five years, in electrochemical sensors based on conducting polymers. Finally, some important remarks and perspectives are presented for this technique as well as summary tables of the performance for the different electrochemical sensors revised.

## 2. Fundamentals of the Electrochemical Sensors

A chemical sensor is a device composed of a recognition element (receptor) coupled with a physicochemical transducer, which transforms chemical information into analytical signals [51,52]. If the recognition system utilizes a biochemical mechanism, it is categorized as a biosensor [53]. These sensors are classified depending on the property that will be analyzed. Thus, electrical, optical, mass, thermal, and electrochemical sensors have been designed [54]. Compared to others, electrochemical sensors are especially attractive because of their remarkable detectability, relative experimental simplicity, and low cost [55]. Electroanalytical sensors commonly work employing a three electrodes cell arrangement, controlled by a potentiostat [56]. The first one is the working electrode (WE) where chemical energy is converted into electrical energy when a redox reaction takes place. The second one corresponds to the reference electrode (RE) that has a well-known redox pair with defined and stable equilibrium potential; it helps to fix or control the difference of potential applied to the WE. The third one is the counter electrode (CE) where the complementary redox semi reaction occurs. The aqueous electrochemical cell is composed of a solution that includes a large amount of supporting electrolyte to improve the ionic conductivity and reduce the migration component in the mass transfer and one or many analytes [57]. Different perturbation signals give us several electrochemical techniques depending on the parameter measured, e.g., potential, current, time, or charge [56]. There are two commonly used electrochemical devices, voltammetric and amperometric sensors [42].

In the voltammetric sensors, analytes in the solution interact onto the surface of the electrode, where the potential can be controlled, undergoing charge transfer (oxidation or reduction) which produces the current that is measured [58]. The current measured has two components in voltammetric measurements: Faradaic current and charging current known as well as non-faradaic current. The faradaic current originates from electron transfer between the analyte and the electrode surface; it is directly proportional to the concentration of the molecule under study [59]. The charging current is not an analytical signal and it is formed when the double layer at the working electrode is charged or discharged by changes into the potential of the electrode [54]. Linear scan voltammetry (LSV) and cyclic voltammetry (CV) uses a perturbation signal consisting of a ramp of potentials [60]. The applied signal for CV is a triangular waveform, where the scan rate is the slope of the curve [61]. The voltammogram is obtained by plotting the response current signal (*i*) versus the potential applied [62] where important parameters are measured such as the anodic and cathodic, peak currents, and peak potentials [63]. The peak current for a reversible system in pure diffusion is described by the Randles–Ševčík Equation (1) as follows:

(1) $$i_{p}=\left(2.69\times 10^{5}\right)n^{3/2}AD^{1/2}Cv^{1/2}$$

where *i_p_* is the peak current (A), *n* are the electrons exchanged in the reaction, *A* is the electroactive area (cm^2^), *D* is diffusion coefficient (cm^2^ s^−1^), *C* is the concentration of the active species (mol cm^−3^), and *v* is scan rate (V s^−1^). The relationship between peak current and concentration of the active species is particularly important in analytical applications and studies of electrode mechanisms. The ratio of peak currents can be significantly influenced by chemical reactions coupled to the electrode process [64]. Different voltammetric techniques, known as pulse techniques, are usually employed to eliminate the charging current and enhance the faradaic current. Differential pulse voltammetry (DPV) and square wave voltammetry (SWV) are quite common techniques for trace analysis [65]. DPV and SWV are techniques where potential pulses are applied upon a staircase waveform ramp potential [66]. After a base potential value is chosen in DPV, the potential increases between pulses with equal increments. The current is immediately measured before the pulse application and at the end of the pulse, then the difference between them is recorded [67]. SWV perturbation signal shows a waveform formed by a series of pulses increasing along a linear baseline where the resulting current is determined by the difference between the current measurement in the forward and backward pulses [68,69]. LSV and CV are commonly used for exploratory purposes, while DPV and SWV are used for quantitative determinations [70,71].

In the amperometric sensors, a constant potential pulse is applied between a reference and a working electrode [72]. If a sufficiently large pulse of potential is applied, the electroactive species interchange electrons, from (reduction) or towards (oxidation) the electrode surface. These electron flows generate the faradaic current [70,73]. Amperometric sensors have to ensure two requirements (i) the transport of the electroactive species must be governed by diffusion, and (ii) the electrode surface must remain constant [74,75]. These sensors display high sensitivity, a wide detection range, short response time, and selectively between several electroactive species in solution [42]. In a chronoamperometry, the obtained current is related to the bulk concentration of the electroactive species according to the Cottrell Equation (2) as follows:

(2) $$i=\frac{nFAD^{1/2}C}{\pi t}$$

where *i* corresponds to the diffusion current (A), *t* is the electrolysis time (s), *n* is the number of electrons involved in the reaction, *A* is the electrode area (cm^2^), *D* is the diffusion coefficient (cm^2^ s^−1^), *F* is the Faraday constant and *C* is the concentration of the electroactive species (mol L^−1^) [76].

## 3. Electrochemical Sensors Based on Conducting Polymers

### 3.1. Neurotransmitters

Biomarkers of the body appeared as a powerful tool to improve the prevention, detection, and treatment of different diseases and degenerative disorders. Neurotransmitters are one of the most important biomarkers because they regulate many functions in cells and tissues. Dopamine (DA) is a molecule that has a fundamental role in cardiovascular, kidney, central nervous, and hormonal system regulation [77]. Abnormal concentrations of DA are related to diseases or disorders such as cancer, Parkinson’s, Huntington’s, dementia [78], and trending to drug dependence [79]. Serotonin (SER) has a strong influence on mood and sleep regulation [80]. Schizophrenia, depression, and other neuropsychiatric disorders, as well as substance use disorders, have been associated with the imbalance of SER [81]. Another important neurotransmitter is epinephrine (EP) which is known as an alert hormone because it boosts the supply of oxygen and glucose to the brain and muscles in emergency situations. Similar to DA, EP levels in the body are related to Parkinson’s disease [36] but it also has therapeutic application for asthma, sepsis, severe allergic, cardiac arrest, and anaphylaxis [82].

#### 3.1.1. Dopamine (DA)

Selective sensors based on polypyrrole (PPy) have been used for DA detection mainly due to environmental stability, good biocompatibility, and high surface area [83]. Furthermore, polypyrrole is easily synthesized and shows higher conductivity in comparison with other conducting polymers [84]. The amine group (–NH–) on the pyrrole ring enhances the capability of this polymer for biomolecular sensing [85] and provides a non-sensitive character to interferences in the solution [86].

**Figure 1:**
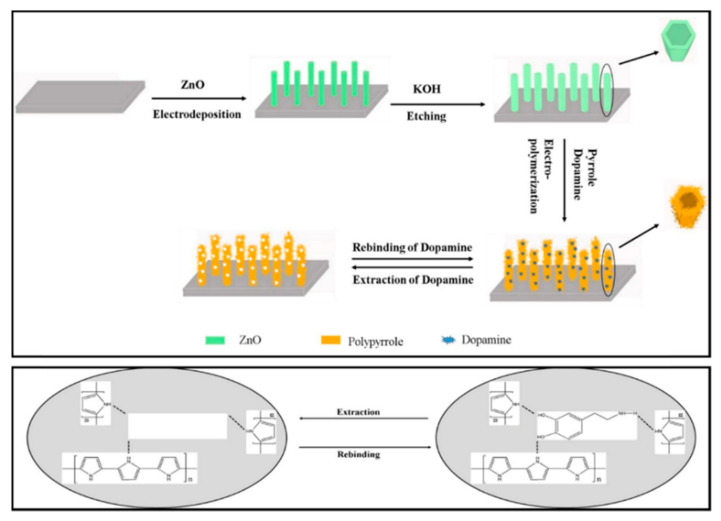
Schematic representation of the molecularly imprinted polymers fabrication process of composite MIPs/ZNTs/FTO glass and its interaction with dopamine. Reproduced with permission from [90]. Copyright 2017 Elsevier B.V.

Polypyrrole films doped with anionic sulfonated β-cyclodextrin (PPy-SβCD) were potentiostatically deposited on platinum electrodes [87]. The obtained films showed a structure with ridges and valleys which generate a ladder-like arrangement. LOD of 1 µM were chronoamperometrically determined for DA at NaCl solutions. Moreover, this modified electrode showed high selectivity for DA due to a strong interaction between cyclodextrin dopant and the protonated DA. A hybrid sensor based on electrochemically reduced graphene oxide and overoxidized electropolymerized polypyrrole (OPPy/ERGO) onto a glassy carbon electrode was made for selective detection of DA [88]. First, reduced graphene was prepared by cyclic voltammetry in a GO/PBS solution at pH 7.4. Then, PPy was potentiodynamically deposited from a pyrrole solution. SEM analysis showed pristine PPy/ERGO deposits had laminated and spherical structures (attributed to PPy). After overoxidation in a NaOH solution, a rough, uniform, and compact thin film was obtained with the incorporation of carbonyl groups. LOD was determined by amperometric measurements resulting in a value of 0.2 μM with a linear response between 0.4 μM and 517 μM. A negatively charged sensor surface enhanced the adsorption of positively charged DA. A similar approach was taken by Demirkan et al. where palladium nanoparticles supported on polypyrrole/reduced graphene oxide (rGO/Pd@PPy NPs) were developed for ascorbic acid (AA), DA, and UA sensing [89]. TEM images of rGO/Pd@PPy NPs nanocomposites showed spherical Pd nanoparticles distributed into the polymeric film. LOD by DPV for AA, DA, and UA were 4.9 × 10^−8^ M, 5.6 × 10^−8^ M, and 4.7 × 10^−8^ M, respectively. This sensor shows electrocatalytic performance, effective electron transfer capability, and better sensitivity because of the synergistic effects of its components. Hybrid composite of molybdenum oxide-based three-dimensional metal-organic frameworks (MOFs) with helical channels combined with polypyrrole (CuTRZMoO_4_@PPy-n) were tested for DA detection by Zhou et al. [83]. Polypyrrole was employed to boost the conductivity of the preset MOF. Structural analysis reveals a coarse, irregular, and circular fringe nanocomposite surface. A LOD of 80 nM was obtained by DPV and a linear range in a PBS pH 2.5 solution from 1 µM up to 100 µM. ZnO nanotubes supported on molecularly imprinted polymers arrays (MIPs/ZNTs/FTO glass) were used for DA detection [90]. Zn nanorods (ZNRs) were deposited by potentiostatic methods onto fluorine-doped tin oxide (FTO). Then, ZNRs were tuned into Zn nanotubes (ZNT) by chemical etching in alkaline solution at low temperatures. Polypyrrole films were electrodeposited from a solution of the monomer, lithium perchlorate, and DA. Finally, the electrode was potentiodynamic cleaned in PBS to oxidize and eliminate the embedded DA. SEM images showed cylindrical ZNT coated with PPy films. High selectivity for DA was assigned to the molecular printing technique (see Figure 1).

**Figure 2:**
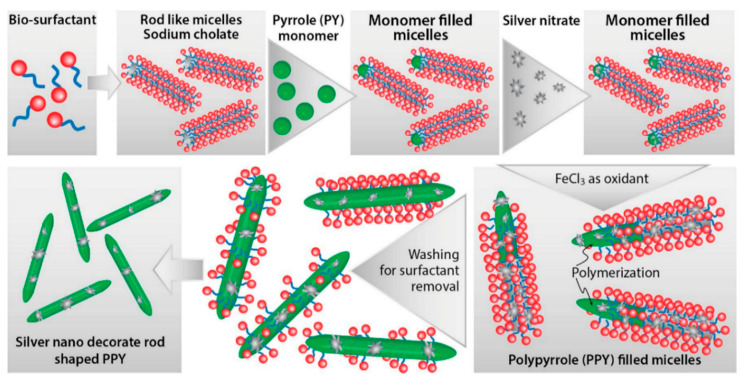
Schematic representation of the fabrication process of composite polypyrrole (PPy)-Ag. Reproduced with permission from [93]. Copyright 2020 Elsevier B.V.

PPy/C#SiO_2_ nanocomposite was synthesized using a mixture of pyrrole and previous manufactured carbon-coated mesoporous SiO_2_ composite (C#SiO_2_) [91]. The deposition of PPy was confirmed using WAXD and FTIR. LOD of 7.6 × 10^−7^ M was determined by DPV within a linear range of 1 × 10^−6^–2 × 10^−4^ M. This electrode showed a small charge-transfer resistance because of the synergetic effect of compounds. Overoxidized polypyrrole/sodium dodecyl sulfate (SDS)-modified multi-walled carbon nanotube (OPPy/SDS-CNT) composites were assembled on gold electrodes by potentiostatic techniques [92]. After polypyrrole co-deposited with SDS and MWCNT, electrodes were overoxidized in a NaOH for generated carboxylic and carbonyl groups in the composite surface. Field emission scanning electron microscopy (FESEM) images showed a rough surface in the pristine deposit due to aggregates of PPy/SDS-CNT which partially disappeared by overoxidation. DA in phosphate buffer solution was detected by DPV showing a linear range from 5 nM to 10 nM and a LOD of 136 pM. The high sensibility of this method is attributed to electrostatic interaction between positively charged DA and negatively charged OPPy/SDS-CNT electrode. The nanocomposite of polypyrrole and silver nanoparticles (PPy-Ag) have been also used for DA sensing [93]. Black solid particles of PPy-Ag nanocomposite were synthesized (see Figure 2) and further studied by SEM and TEM. The PPy-Ag showed a rod-like structure with embedded spherical Ag nanoparticles. A detection limit of 50 pM and linear range from 0.05 nM to 3 nM was obtained for DA using LSV in a solution of PBS at pH 7. The current signal remained almost the same after five measurements with an interval of 25 min each in 0.05 nM DA. A better electroactive surface that facilitates the tunneling of electrons within the redox couple is responsible for this high sensitivity. Additionally, a biocompatibility assay was performed in mouse fibroblast cells exhibiting low toxicity.

Polyaniline (PANI) appears as one of the most used CP materials for a sensor assembly. PANI presents interesting properties such as stability, flexibility, good electrical, and optical properties [94]. It has –NH– functional groups in its structure that improve the adsorption of analytes [95]. The low cost, high yield manufacturing process [96] and the possibility to switch between the insulating and conducting phases by acid/base process [97] makes PANI one of the most versatile CPs for application in the sensing field.

**Figure 3:**
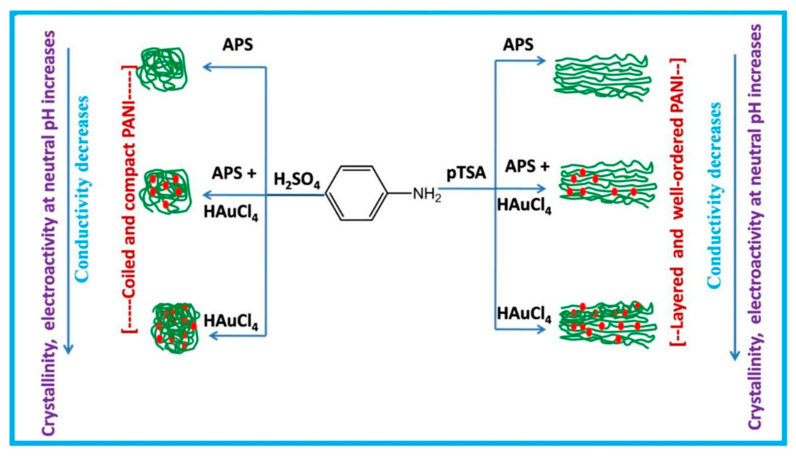
Schematic representation of different synthetic pathways for manufacturing the composite polyaniline-p-toluene sulphonic acids PANI-pTSA. Reproduced with permission from [98]. Copyright 2019 Elsevier B.V.

Polyaniline-gold nanocomposite (PANI-Au) were fabricated as DA sensors by two different combined acidic and oxidative doping pathways as shown in Figure 3 [98]. Ammonium persulphate (APS) and chloroauric acid (HAuCl_4_) were employed as oxidant agents while p-toluene sulphonic acids (pTSA) and sulfuric acid were used as protonic acid dopants. SEM images showed PANI-H_2_SO_4_ had dense nature while PANI-pTSA had layered morphology with high porosity. Spherical Au nanostructures were deposited over polymeric films. PANI-pTSA@Au sensors gave a LOD of 5.25 μM within a linear range of 7–100 μM. These sensors generated well-defined signals for DA in the presence of inferences.

**Figure 4:**
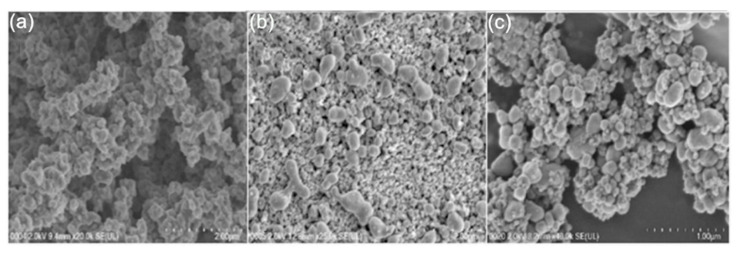
SEM images of (**a**) PANI, (**b**) poly-β-CD, and (**c**) poly-β-CD (f-MWCNTs)/PANI composite. Reproduced with permission from [99]. Copyright 2019 Elsevier B.V.

Polyaniline deposited over glassy carbon has been also used as support for the electropolymerization of beta-cyclodextrin (β-CD)/hydroxyl functionalized multi-walled carbon nanotubes (f-MWCNTs) in PBS solution at pH 7 [99]. Poly-β-CD(f-MWCNTs)/PANI nanocomposite showed a porous granular morphology related to the PANI and poly-β-CD microstructures (see Figure 4) resulting in high surface areas. LOD of 0.0164 μM was determined by DPV. The sensitivity obtained for this electrode was ascribed to the high porosity and high surface area. A sensor based on a derivative of poly (o-methoxyaniline)-gold (POMA-Au) nanocomposites showed a LOD of 62 nM within a linear range from 10 µM to 300 µM for DA [100]. POMA provided a large surface area while Au nanoparticles high electrical conductivity. Poly (aniline-co-o-anisidine)/graphene oxide nanocomposites coated with Au nanoparticles (AuNPs/PANI-co-PoAN/GO) was also fabricated for DA sensing applications [101]. A copolymer of aniline and o-anisidine was synthesized by adding ammonium persulfate to a solution of hydrochloric acid containing both monomers and GO. Au electrodes were dipped coated into a PANI-co-PoAN/GO solution in chloroform, followed by potentiodynamic deposition of Au nanoparticles from a KCl/HAuCl_4_ solution. LOD for DA using SWV was 33.4 nM within a linear range of 5–100 µM. This sensor showed a fast electron transfer and high surface area due to the presence of gold nanoparticles.

Poly (N-(Naphthyl) ethylenediamine dihydrochloride) nanofibers onto anodized glassy carbon electrodes (PNEDA/AGCE) were developed as DA electrochemical sensors by Rahman et al. [102]. DPV was employed for DA determination, with concentrations in the range of 0.1–100 µM and a LOD of 70 nM. DFT calculations showed a strong H-bonding interaction between the free –NH_2_ groups of PNEDA and oxidizable –OH groups of DA resulting in a good sensitivity for this sensor. Graphene/poly (o-phenylenediamine) (GP/PoPD) was potentiodynamically deposited onto pencil graphite electrodes (PGE) from lithium perchlorate, o-phenylenediamine and graphene solution [103]. LOD of 0.16 nM was obtained by SWV within a linear range of 1.0 nM–150 μM. This low LOD was ascribed to a high electroactive surface area and fast electron transfer. A highly-selective sensor for DA was developed using poly-4-Amino-6-hydroxy-2-mercaptopyrimidine (Poly-AHMP) film over a glassy carbon electrode [104]. A highly rough and porous surface was observed in SEM images of the film resulting in an increased active surface area of the electrode. This sensor showed a LOD of 0.2 µM within a linear range from 2.5 µM to 25 µM employing DPV.

Different polythiophene derivatives have shown potential in the fabrication of electrochemical sensors [105,106] among them poly (3,4-ethylenedioxythiophene (PEDOT) is considered a top choice due to its high electrical conductivity which is in the order of magnitude of silver and copper [107], huge optical transparency at visible light and better room stability than PPy [108]. Furthermore, PEDOT presents extraordinary redox reversibility [109] which provides antifouling properties that expand the using lifetime of the polymer film [108]. PEDOT has also the advantage of easy synthesis [110], and generation of deposits with a low tensile module enduring constant mechanical deformation generally related to biological applications [111].

**Figure 5:**
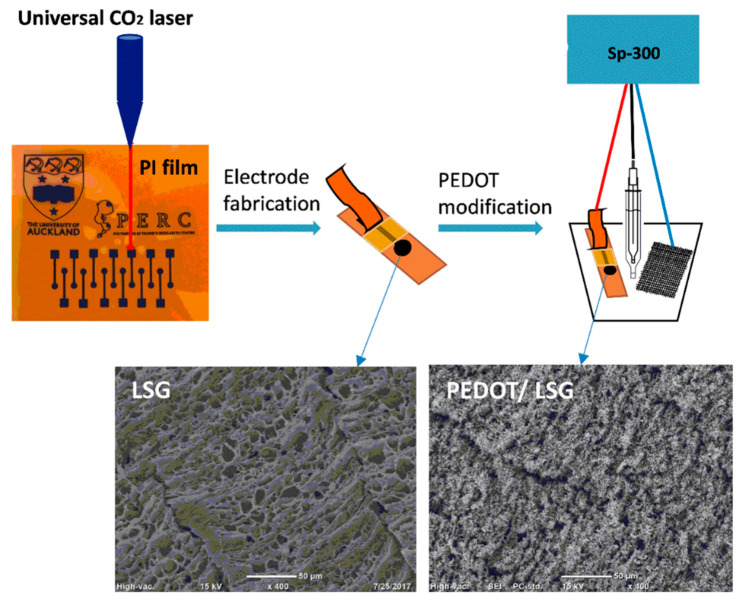
Schematic representation of the fabrication process of PEDOT-Modified Laser Scribed Graphene (PEDOT-LSG) electrodes, and micrographs showing the morphology of LSG and PEDOT/LSG film. Modified with permission from [112]. Copyright 2018 Elsevier B.V.

PEDOT-Modified Laser Scribed Graphene (PEDOT-LSG) electrodes were used as an electrochemical sensor for DA [112]. LSG had a regular and smooth flake structure. After PEDOT electropolymerization a 3D porous network structure remains (see Figure 5). A LOD of 0.33 µM with a linear range of 1–150 µM was obtained by DPV in PBS solution at pH 7. The sensitivity of this sensor was related to the rapid electron transport properties of porous graphene combined with the electrocatalytic activity of PEDOT deposit.

**Figure 6:**
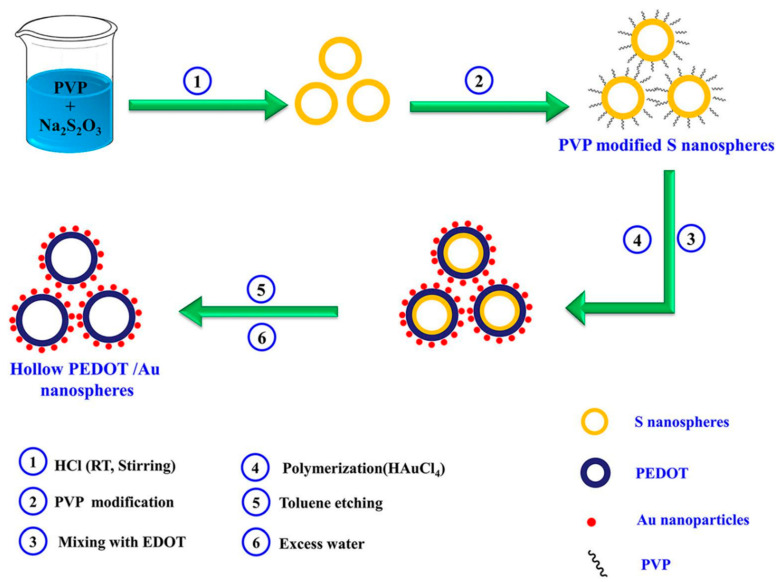
Schematic representation of the fabrication process of PEDOT/Au composites. Reproduced with permission from [114]. Copyright 2017 Elsevier B.V.

Sandoval-Rojas et al. fabricated poly (3,4-ethylenedioxythiophene) doped with a bis(pyrazolyl)methane disulfonate sensors (PEDOT/LSA) for DA detection [113]. This electrode was synthesized over a glassy carbon electrode using potentiodynamic voltammetry from an EDOT and sodium salt of bis(3,5-dimethyl-4-sulfonate-pyrazole-1-yl)methane solution in acetonitrile/deionized water. The dopant produced large globular structures on the polymer surface. A LOD of 0.26 µM within a linear range from 0 μM to 5 μM was obtained using DPV. Monodispersed poly (3,4-ethylenedioxythiophene)/gold hollow nanospheres (PEDOT/Au) electrodes were also designed for DA sensing [114]. The composite was synthesized over a glassy carbon electrode in the aqueous phase. Hollowed nanospheres template was precipitated from a stirred Na_2_S_2_O_3/_PVP solution. Then PEDOT/Au hollow nanospheres were produced by stirring PVP modified sulfur nanospheres in an EDOT/HAuCl_4_ solution (see Figure 6). SEM micrographs revealed a 3D globular structure with a size of 300 nm to 1000 nm. Linear range and LOD values of 0.15 μM to 330 µM and 70 nM respectively, were reported by using DPV. The excellent performance of this electrode was ascribed to the fast electron charge transfer kinetics of this composite.

**Figure 7:**
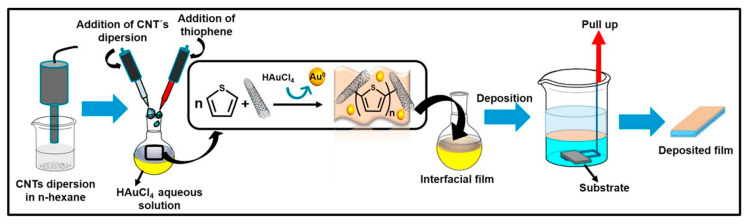
Schematic representation of the fabrication process of PT/Au/CNT electrodes. Reproduced with permission from [120]. Copyright 2019 Elsevier B.V.

Composites of multi-walled carbon nanotubes and nanoceria-poly (3,4-ethylenedioxythiophene) (MWCNTs/CeO_2_-PEDOT) has been used for DA detection [115]. PEDOT films agglomerated into sphere-like grains preserving this structure in the composite with diameters of the particle between 200 nm and 450 nm. A LOD of 30 nM within a linear range of 0.1–10 µM was determined by DPV measurements. Poly (3,4-ethylenedioxythiophene)/reduced graphene oxide/manganese dioxide modified glassy carbon electrodes (PrGO/MnO_2_) were built for simultaneous detection of DA, UA, and AA [116]. After potentiodynamic electrodeposition of PrGO on a glassy carbon electrode, MnO_2_ was deposited using a solution of KMnO_4_ and H_2_SO_4_. PEDOT appears as a granular film deposited over rGo. The MnO_2_ is observed as small particles onto PrGO. The sensor structure provided a high surface area which increases the sensitivity. This composite shows high electrocatalytic activity that generated a well-separated oxidation potential of UA, DA, and AA. Simultaneous detection gave LOD values of 50 nM (UA), 20 nM (DA), and 1.0 µM (AA) in PBS solution at pH 6. Poly (3,4-ethylenedioxythiophene) doped with ionic liquid (1-ethyl-3-methylimidazolium bis(trifluoromethylsulfonyl)imide) on glassy carbon electrode (PEDOT/IL/GCE) has been also used as biofouling resistant DA electrode showing porous microstructure, high electrical conductivity, and good stability [117]. LOD and linear range values of 33 nM and 0.2 μM to 328 μM, respectively, were found for DA sensing in presence of proteins such as bovine serum albumin (BSA), human serum albumin (HSA), and chicken egg white lysozyme (LZM). Spin coated poly (3,4-ethylenedioxythiophene):polystyrene functionalized with beta-cyclodextrin sensors (CD-f-PEDOT:PSS) for DA and catechol were fabricated by Qian et al. [118]. AFM images showed PEDOT: PSS surface changes by treatment with H_2_SO_4_ from polymer particles to entangled wires boosting the electrical conduction. The obtained LOD and linear range were 9.6 nM and 50 nM to 200 µM, respectively, by using DPV into a PBS buffer solution at pH 7.4. Highly sensitive DA sensors were developed by Pananon et al. using a nanocomposite made of gold nanoparticles, graphene (GP), and poly (3,4-ethylenedioxythiophene):polystyrene sulfonate (AuNP-GP-PEDOT:PSS/GCE) using a green synthetic method [119]. SEM images proved a uniform distribution of gold nanoparticles onto the surface. This sensor shows one of the lowest detection limits for DA of 100 pM with linear dynamic range from 1 nM to 300 µM related to an increased surface area, high catalytic activity of AuNP and a superior conductivity of GP and PEDOT:PSS. Moreover, thin polythiophene films composed with gold nanoparticles and carbon nanotubes (PT/Au/CNT) were synthesized by liquid–liquid interfacial reaction [120]. The construction of this composite required an aqueous mixture of dispersed CNT, HCl, HAuCl_4_.3H_2_O, and thiophene, in a molar relation 1:1 with HAuCl_4_. Modified electrodes were self-assembled by putting a substrate (silicon, quartz or glass) into a stirred solution for 4.5 h (see Figure 7). This method resulted in a sensor with a LOD of 0.69 μM for DA using DPV. A relative standard deviation (RSD) of 3.96% was obtained using three modified electrodes which performed 25 measurements each one. These results pointed out an enhanced charge transfer related to the presence of CNT.

**Figure 8:**
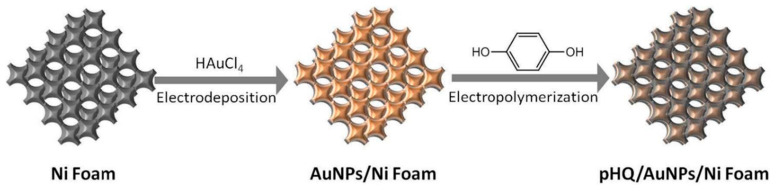
Schematic representation of the fabrication process of pHQ/AuNPs over Ni Foam. Reproduced with permission from [125]. Copyright 2017 Elsevier B.V.

Unconventional conducting polymers have also been used for electrochemical sensing. Poly (Sudan III) was potentiodynamically deposited over carbon paste electrodes (PS/MCPE) from a solution containing NaOH and Sudan III [121]. SEM images showed irregularly shaped graphite flakes at the surface. A LOD of 9.3 µM with a linear range of 10–90 µM was determined by DPV. Polyphenol red film on glassy carbon electrode was used for detection of DA and acetaminophen [122]. Potentiodynamic polymerization of this molecule is possible through the quinone methide group. Sensing experiments were carried out in PBS solutions at different pH values. A LOD and linear range for DA were 1.6 μM and 20–60 μM, respectively. The value of the catalytic rate constant (8.45 × 10^2^ M^−1^ s^−1^) demonstrates that p-PhR/GCE has a catalytic oxidative reaction for DA. Poly (procaterol hydrochloride) modified glassy carbon electrodes (p-ProH/GCE) were used for DA and UA detection in human serum [123]. These sensors were built by a potentiodynamic method in a PrOH solution on glassy carbon electrodes. Modified electrodes showed a high affinity for DA with a LOD value of 0.3 µM within a linear range of 1–100 µM by square wave voltammetry in PBS at pH 5. Composites of poly (glyoxal-bis(2-hydroxyanil)), amino-functionalized graphene quantum dots, and MnO_2_ nanoclusters were deposited over glassy carbon electrodes (GCE/PGBHA-afGQDs-MnO_2_) for vitamin B12 and DA sensing [124]. SEM images displayed a rough and dense film with GQDs clusters made of particles with a size less than 50 nm which increased the roughness hence the surface area and electroconductivity resulting in LOD of 50 nM for DA by DPV. Poly (hydroquinone)/gold nanoparticles/nickel foam (pHQ/AuNPs/NF) were used for DA sensitive detection [125]. First, gold nanoparticles were deposited from a solution containing HAuCl_4_, over previously cleaned nickel foam, by potentiostatic methods. Then potentiodynamic polymerization of hydroquinone was performed in phosphate-buffered solution at pH 5 (see Figure 8). Micrographs showed a porous 3D network structure of NF with a rough surface due to the deposited pHQ/AuNPs. These modifications of Nickel foam provide a large surface area and high conductivity. Determination of DA was made using DPV resulting in a LOD and linear range of 41.9 nM and 1.0 × 10^−7^ M to 1.0 × 10^−5^ M, respectively.

**Figure 9:**
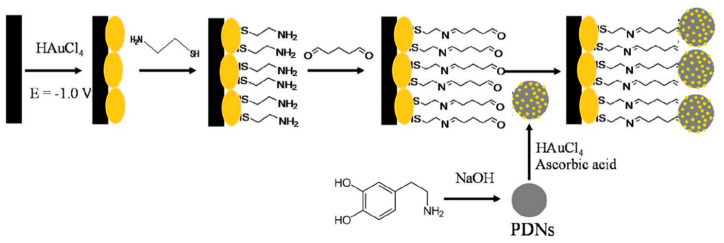
Schematic representation of the fabrication process of Au-PDNs (polydopamine nanospheres) electrodes. Reproduced with permission from [128]. Copyright 2019 Elsevier B.V.

Simultaneous detection of AA, DA, and UA was performed using a sensor based on electrochemically reduced graphene oxide-poly (eriochrome black T)/gold nanoparticles (ERGO-pEBT/AuNPs) modified glassy carbon electrodes [126]. FESEM technique showed a uniformly rough composite surface with Au nanoparticles homogeneously distributed leading to LOD values of 9.0 nM for DA. Carboxylic acid functionalized multi-walled carbon nanotubes/polytoluidine blue over glassy carbon electrodes (MWCNTs-COOH/Poly (TB)/GCE) showed high sensitivity to DA (LOD = 0.39 nM) related to the high surface area of the net-structure MWCNTs-COOH and the electrocatalytic activity of polymer [127]. Arroquia et al. fabricated self-assembled gold-decorated-polydopamine nanospheres (Au PDNs) for simultaneous detection of AA, DA, UA, and tryptophan [128]. First, synthesis of polydopamine nanospheres (PDNs) involved 3 h of stirring in DA hydrochloride/NaOH solution at 50 °C. Next, the suspension of PDNs was mixed with HAuCl_4_ and AA to get Au nanospheres (Au-PDNs). Finally, Au-PDN composite was deposited onto a screen-printed carbon electrode previously modified with gold nanoparticles, cysteamine, and glutaraldehyde (see Figure 9). Electronic microscopy showed a homogeneous distribution of Au-PDN nanospheres onto modified electrodes resulting in high surface areas with an improved charge transfer process. A remarkable LOD of 0.1 nM was determined for DA with a linear range from 1 µM to 160 µM by DPV. This electrode was employed for extended periods (70 work hours and 200 measurements) showing no loss in the intensity of the signal.

Dopamine sensors have shown high sensitivity due to the high surface area of the conducting polymer films and small charge/transfer resistance generated by materials such as metal nanoparticles, RGO, and MWCNT. Besides, these materials have the role to boost the negative charge of the electrode surface establishing a strong electrostatic interaction with the positively charged analyte. The fact that dopamine is required to be detected usually in the presence of UA and AA represents a big challenge for researchers; however, this issue has been tackled by using molecular imprinted polymers as well as voluminous molecules such as B/cyclodextrin. In this way, a strong and specific interaction is generated with the analyte resulting in the separation between the oxidation potential of DA and the interferences.

#### 3.1.2. Epinephrine (EP)

**Figure 10:**
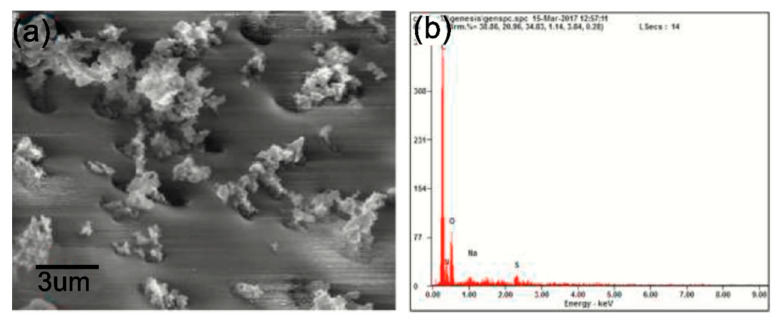
SEM analysis of EB-PPy-BSA hybrid structure (**a**) micrograph, and (**b**) EDS spectrum. Modified with permission from [129]. Copyright 2018 Elsevier B.V.

Electron beam irradiated polypyrrole nanospheres/bovine serum albumin onto glassy carbon electrodes (EB-PPy-BSA/GCE) were used for EP and L-tyrosine detection [129]. A mixture of methyl orange, FeCl_3,_ and pyrrole was used to prepare polypyrrole nanospheres which were treated with electron beam radiation. Polypyrrole nanospheres and bovine serum albumin solution were sonicated for 2 h followed by drop-casting onto a glassy carbon electrode. SEM revealed that PPy nanospheres were embedded into a porous structure of BSA (see Figure 10). SWV was used for analyte determination obtaining a LOD of 7.1 nM. Seven different electrodes were potentiodynamically tested in solutions containing 200 uM EP giving an RSD of 5.3%. The use of BSA provided a large surface area, excellent structure stability, rich pore channels, and a redox mediator role. Tea and chicken extracts were evaluated with this sensor giving promising results for biological and healthcare applications. Ghanbari and Hajian reported the fabrication of gold nanoparticles/Zinc oxide/polypyrrole/reduced graphene oxide nanocomposite (Au/ZnO/PPy/RGO) on glassy carbon electrode for detection of AA, EP, and UA [130]. Polypyrrole deposits appeared as nanofibers onto RGO surface. LOD of 58 nM and linear range from 0.6 µM to 500 µM was obtained by DVP in PBS solution at pH 7. This sensor was tested in a human serum sample giving recovery values above 97%.

Three-dimensional mesoporous polymeric graphitic-C_3_N_4_/polyaniline/CdO nanocomposite (mpg-C_3_N_4_/PANI/CdO) was electrochemically synthesized by Bonyadi et al. for simultaneous sensing of EP, paracetamol, mefenamic acid, and ciprofloxacin [131]. FESEM exposed a nanofiber-like structure of PANI deposited over the 3D structure made by C_3_N_4_ resulting in a tremendous increase of the electrode surface area. A LOD of 11 nM and two linear ranges from 0.05 µM to 80 µM and from 100 µM to 1000 µM were obtained for EP using DPV in PBS solution at pH 7.4. A 98.9–102.6% recovery for EP was obtained in human blood serum samples. Polyaniline nanocomposite films have also been doped with TiO_2_ and RuO_2_ nanoparticles on multi-walled carbon nanotubes (MWCNT-PANI-TiO_2_ and MWCNT-PANI-RuO_2_) for EP sensing [132]. TiO_2_ or RuO_2_ nanoparticles, MWCNT, and PANI were dissolved in DMF followed by sonication for 24 h to generate the nanocomposite. This suspension was drop coated onto Au bare electrode. PANI/MWCNT fibers formed tube-like structures with TiO_2_ and RuO_2_ spherical shaped particles that increased the porosity of the composite and its surface area_._ EP determination was performed using DPV in a PBS solution at pH 7 with concentrations from 4.9 µM to 76.9 µM. LODs of 0.16 µM for MWCNT-PANI-TiO_2_ and 0.18 µM for MWCNT-PANI-RuO_2_ were obtained. Both sensors were tested in an EP injection given more than 99% recovery. PANI derivatives such as molecular imprinted poly (3-aminophenylboronic acid) has also been composited with multi-walled carbon nanotubes (PAPBA(MIPs)/MWCNTs) onto glassy carbon electrode for EP sensing showing a LOD of 35 nM [133]. Molecular printing provides selectivity to distinguish EP from potential inferences. Following a similar strategy, molecularly imprinted poly 3-thiophene boronic acid (P3-TBA)/gold nanoparticles (MIP/AuNP) composite was developed by Liu and Kan obtaining a selective sensor of EP from its analogs resulting in a LOD of 76 nM by DPV in PBS solution at pH 7 [134]. This sensor had double recognizing ability due to (i) reversible covalent interaction between boronic acid of 3-TBA and cis-diol of EP, and (ii) size and shape complementarity between template molecules and imprinted sites. A 90.6% to 103.5% recovery was obtained in a real EP injection using this sensor.

A glassy carbon electrode modified with gold nanoparticles into poly-fuchsine (FA) acid film (poly (FA)/AuNP/GCE) was used for simultaneous detection of AA, EP, and UA [135]. The poly (FA) was deposited by CV from a solution of fuchsine acid and NaOH. Then, AuNPs were electrodeposited by immersing the electrode into a solution of HAuCl_4_ and KNO_3_. This electrode had a LOD of 10 nM for EP and 9 nM for AA in a buffer solution at pH 3. This method was proved in real samples using the standard addition method obtaining recovery values of 87.0% in hydrochloride injection and 102.0% in urine for EP. Potentiodynamic generation of poly (brilliant cresyl blue) on graphene/glassy carbon electrode (PBCB/graphene/GCE) was employed for detection of EP resulting in a LOD of 0.24 µM by CV in PBS solution at pH 7 for an EP concentration ranging from 1 µM to 1000 µM [136].

#### 3.1.3. Serotonin (SER)

**Figure 11:**
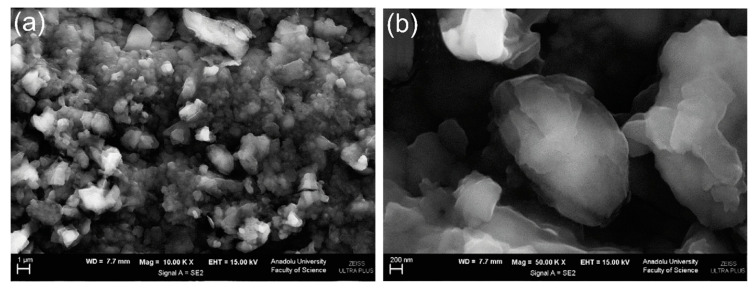
SEM image of p(P3CA)/PGE surface in a magnification of (**a**) 10,000× and (**b**) 50,000×. Modified with permission from [137]. Copyright 2015 Elsevier B.V.

Poly (pyrrole-3-carboxylic acid) modified pencil graphite electrodes (p(P3CA)/PGE) were electrochemically generated for SER sensing in biological samples [137]. SEM micrographs showed cauliflower-like structures of P3CA (see Figure 11) that increase the surface area in comparison with a flat surface of the bare GE. Adsorptive differential pulse stripping voltammetry (AdSDPV) was applied for the determination of SER concentrations from 0.01 µM to 1.0 μM in a PBS solution at pH 5 resulting in a LOD of 2.5 nM. This sensor was tested in blood serum and urine samples giving a 97.7% to 100.6% recovery and 93.8% to 97.4% recovery, respectively.

**Figure 12:**
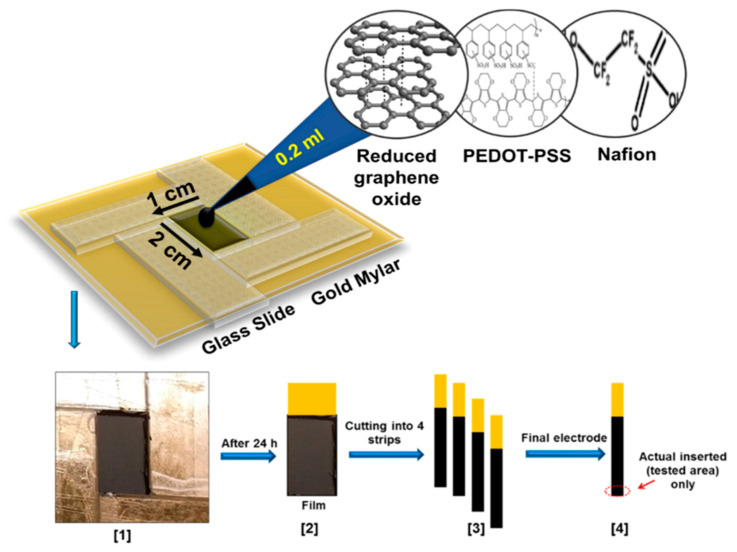
Schematic representation of the casting process of rGO−PEDOT/PSS-nafion composite onto gold mylar substrates. Reproduced with permission from [141]. Copyright 2019 American Chemical Society.

Ran et al. fabricated a poly (p-amino benzene sulfonic acid), multi-walled carbon nanotubes, and chitosan nanocomposite on glassy carbon sensor (MWCNTs–CS–poly (p-ABSA)/GCE) for SER detection [138]. Poly (p-ABSA) film was potentiodynamically obtained over GCE followed by drop-casting of MWCNTs–CS suspension. DPV sensor for SER displayed a linear range of 0.1–100 μM and a LOD of 80 nM in PBS buffer solution at pH 7. A recovery between 97% and 98% was obtained in human blood serum. A similar monomer derivative was used for the construction of a graphene (GR)/poly 4-amino-3-hydroxy-1-naphthalenesulfonic acid-modified sensor onto a screen-printed carbon (GR/p-AHNSA/SPCs) for simultaneous detection of DA and SER [139]. FE-SEM micrographs exposed that p-AHNSA was deposited over SPC generating nano-rod shape structures interconnected by GR resulting in large surface areas with high electrocatalytic activity. SWV sensor showed a LOD of 3 nM for the SER concertation range of 0.05–150 μM in a PBS solution at pH 7.4. This sensor was used for the determination of SER in plasma and urine obtaining recovery values of 98.1% to 101.2%. The sensor can be used up to 3 times without significant loss in the current response. A well-known pH indicator has also been used for the fabrication of nanocomposites based on poly (bromocresol green), iron oxide nanoparticles, and multiwalled carbon nanotubes (Fe_3_O_4_–MWCNT–poly (BCG) for the detection of SER [140]. This DPV sensor showed a LOD of 80 nM with a linear range of 0.5–100.0 μM in PBS solution at pH 7. A human blood serum sample was used for testing this sensor which provided recovery values of ca. 93%. Reduced graphene oxide/poly (ethylene-3,4-dioxythiophene)/poly (styrene sulfonic acid)/nafion (rGO−PEDOT/PSS-nafion) films were drop-cast by Al-Graiti et al. to detect SER (see Figure 12) [141]. SEM images showed PEDOT/PSS avoid the restacking of rGO resulting in a GO−PEDOT/PSS smooth film. This sensor allowed the simultaneous detection of SER and DA displaying a LOD of 0.1 μM and linear range of 1 to 10 μM for SER by employing DVP in PBS solution at pH 7.4.

Chung et al. designed a DA and SER sensor based on palladium complex Pd(C_2_H_4_N_2_S_2_)_2_ anchored to poly-2,2:5,2-terthiophene-3-(p-benzoic acid) on AuNPs decorated reduced graphene oxide substrates (AuNPs@rGO/pTBA-Pd(C_2_H_4_N_2_S_2_)_2_) [142]. After drop-casting AuNPs@rG onto a screen-printed carbon electrode, pTBA was electrodeposited over the modified working electrode by CV. Activated COOH groups allowed the immobilization of the Pd(C_2_H_4_N_2_S_2_)_2_ on the polymer layer by covalent bond formation. A LOD of 2.5 nM was found by SWV in a buffer solution at pH 7.4. This sensor was used for the quantification of SER in breast cancer cells (MCF-7) by standard addition method obtaining recoveries from 97.2% to 103.8%.

### 3.2. Uric Acid (UA)

UA levels in the human body provide information about the metabolic alterations or diseases such as metabolic syndrome, hypertension, kidney injury, and cardiovascular problems [143] because it is the final product of different metabolic pathways [5].

**Figure 13:**
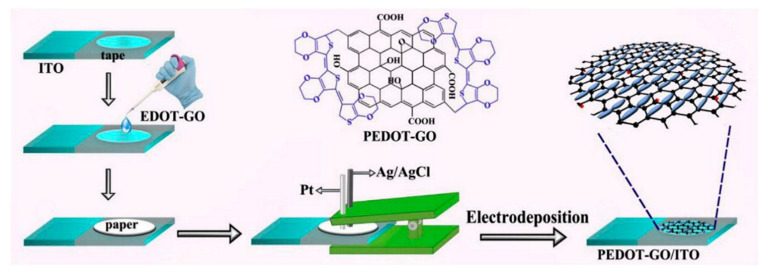
Schematic representation of the fabrication process of PEDOT/GO/ITO electrodes. Modified with permission from [148]. Copyright 2019 Elsevier B.V.

A composite of polytetraphenylporphyrin, polypyrrole, and graphene oxide (p-TPP/PPy/GO) deposited onto glassy carbon electrode was used for detection of UA resulting in a LOD of 1.15 μM with a linear range of 5–200 μM by DPV in PBS solution at pH 7 [144]. P-TPP was used for boosting the electrocatalytic activity towards the oxidation of the analytes. α-Fe_2_O_3_/polyaniline nanotubes (α-Fe_2_O_3_/PANI NTs) were synthesized by Mahmoudian et al. for UA sensing [145]. Polyaniline nanotubes were fabricated from a solution of acetic acid, methanol, aniline, and ammonium persulfate by static synthesis for 10 h. Then, α-Fe_2_O_3_/polyaniline nanocomposite was assembled by stirring a solution of FeSO_4_.7H_2_O and polyaniline nanotubes. TEM and FESEM allowed confirming the formation of PANI NTs with the presence of α-Fe_2_O_3_ spherical and hexagonal nanoparticles that increased the electrode surface area. A DPV sensor was used for UA determination in concentrations from 0.01 μM to 5 μM in PBS solution at pH 7 obtaining a LOD of 38 nM. UA was determined in a real urine sample giving recovery values between 98.58% and 101.98%. A sensor based on functionalized polyaniline derivatives of nanostructured polyortho-methoxyaniline/multi-wall carbon nanotube onto a graphite paste electrode (POMANS-MWCNT/GPE) was used for simultaneous detection of UA and folic acid [146]. A LOD of 0.157 μM and a linear range of 0.6–52 μM was determined for an LSV sensor in PBS solution at pH 6. This electrode was tested in urine and blood serum samples given recovery values higher than 99.6%. A sensitive sensor based on over-oxidized poly (3,4-ethylenedioxythiophene) nanofibers modified pencil graphite (Ox-PEDOT-nf/PGE) was developed for UA detection resulting in a detection limit of 1.3 nM and a linear range of 0.01–20 μM by DPV in PBS at pH 2 [147]. UA was sensed in urine and blood serum samples by standard addition method giving recovery values from 104% to 107%. Huang et al. synthesized poly (3,4-ethylenedioxythipohene)/graphene oxide composites onto ITO electrodes (PEDOT/GO/ITO) for UA determination into saliva [148]. Figure 13 shows the fabrication procedure for this paper-based electroanalytical device. After adding EDOT-GO suspension on ITO substrate, a potentiostatic polymerization was performed in a thin electrochemical cell resulting in a porous film. SEM showed PEDOT-GO films as porous and rough networks. A DPV sensor displayed a LOD of 1.3 nM and a linear range from 2 μM to 1000 μM in buffer solution at pH 6.8.

**Figure 14:**
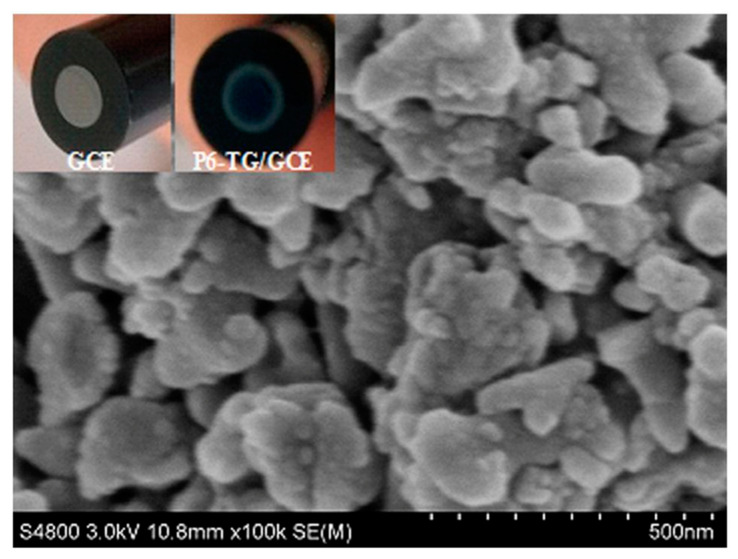
Micrograph of poly (6-thioguanine) film (P6-TG) deposited over glassy carbon electrode. Reproduced with permission from [152]. Copyright 2015 Elsevier B.V.

Molecular imprinted poly (2-amino-5-mercapto-1, 3, 4-thiadiazole) (PAMT) and reduced graphene oxide (MIP/RGO) composite was used for simultaneous determination of UA and tyrosine obtaining a LOD of 3.2 nM for UA by DPV in PBS at pH 5 [149]. This sensor was tested in urine and serum showing recoveries between 94.0% and 106.0%. MIP/RGO sensor was tested 11 consecutive times in 0.4 µM UA resulting in an RSD of 4.23%. Poly (sulfosalicylic acid) and carboxylated graphene modified glassy carbon electrode (PSA/ERCG/GCE) sensor was employed for isoniazid and UA sensing [150]. A DPV sensor gave a LOD of 12 nM for UA in ammonia buffer solution at pH 9.0. Taei et al. fabricated a gold-nanoparticles/poly-Trypan Blue modified glassy carbon electrode (AuNPs/poly-TrB/GCE) for determination of cysteine (Cys), UA, and tyrosine (Tyr) [151]. After potentiodynamic deposition of polymeric film onto the GCE, gold nanoparticles were deposited from AuNPs suspensions by chronopotentiometry. The polymeric films appeared as effective support for AuNPs according to SEM images. A DPV sensor gave a LOD of 70 nM and a linear range from 1 μM to 550 μM for the sensing of UA in PBS solution at pH 3. A film of poly (6-thioguanine) on a glassy carbon electrode (P6-TG/GCE) was electrogenerated by Lan and Zhang for simultaneous detection of DA, UA, xanthine (XA), and hypoxanthine (HXA) [152]. SEM images showed a rough polymeric film (see Figure 14) providing an increased effective surface area of the electrode. A LOD of 60 nM and a linear range from 2 µM to 1600 µM was determined for UA by DVP in PBS solution at pH 7. UA was determined in real samples of urine and blood serum showing recoveries > 98%.

### 3.3. Ascorbic Acid (AA)

**Figure 15:**
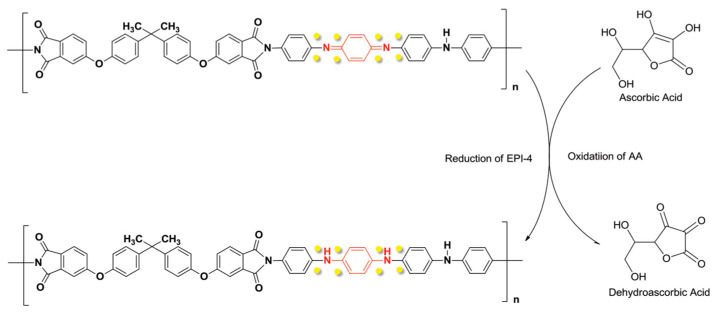
Scheme of EPI-4 structure used into a CPE to determinate AA. Yellow points represent gold nanoparticles. Reproduced with permission from [155]. Copyright 2017 Elsevier.

Shalini A. et al. reported a nanocomposite of polypyrrole with cellulose for AA sensing [153]. The polymer was grafted on cellulose by homogeneous oxidation of the monomer with ammonium peroxydisulfide, then PPy@C was immobilized onto GCE. SEM images showed spherical particles of PPy with 100–200 nm size, coating cellulose. Voltammograms of AA in aqueous solution at pH 7.0 shows oxidation electrocatalytic attributed to a large area of the nanocomposite. A LOD of 75 μM was chronoamperometrically determined for AA at aqueous solutions. Additionally, this modified electrode achieved recoveries ranging between 97.1% to 102.9% for real samples of commercial fruit juices of papaya, tomato, and orange. A layer-by-layer modification of GCE was presented by Fang Y. et al. [154]. After potentiostatic deposition of nickel on glassy carbon electrode, CNTs were grown by catalytic chemical vapor deposition (CCVD) followed by PANI potentiodynamic polymerization from aqueous aniline solution. A three–dimensional network structure was observed by SEM analysis. Voltammograms of hexacyanoferrate(III) with the final modified surface exhibits a peak current increase of ten with respect to bare glassy carbon electrode, due to the enlarged surface area caused by the conducting polymer and the carbon material. AA was determined by DPV measurements obtaining a LOD of 0.1 μM, mol·L^−1^, and recoveries of 97.4–101.2%. In addition, this sensor showed no loss of electroactivity after 30 continuous CVs in 0.2 mM of AA. Two organo-soluble polyimides were synthesized to overcome common problems of commercial conducting polymers as solubility and processing [155]. Electroactive polyimides EPI-3 and EPI-4 were immersed into a HAuCl_4_ aqueous solution for gold nanoparticle formation. Voltammograms of 2 mM AA with different composites shown a peak current for EPI-4 higher in a factor of ten with respect to EPI-3 signal indicating that EPI-4, a longer conjugated structure of diamine, has higher redox capability (see Figure 15). For AA determination EPI-4 was mixed with graphite powder and paraffin oil to prepare a carbon paste electrode. A LOD of 18.5 μM was determined by chronoamperometry within a linear range of 1.0 × 10^−5^ M to 1.0 × 10^−3^ M.

A poly (amido amine) dendrimer, silver nanoparticles, and multi-walled carbon nanotubes composite with poly (neutral red) (PAMAM/AgNPs–MWCNT/PNR film) prepared on a paraffin wax impregnated graphite electrode was reported by Lackshmi C. et al. [156]. A linear range for AA signal was obtained from 2.0 × 10^−7^ M to 2.5 × 10^−3^ M with a LOD of 53 nM with DPV at optimal pH of 4.0. The reproducibility was determined for 60 days measuring 81.5 mM of AA every 5 days. The sensor retained 94% of the initial response. The best performance of this sensor is due to the large surface achieved by a combination of the carbon materials, metallic particles, and conducting polymer. Glassy carbon surface was modified by potentiodynamic polymerization of EDOT into a propylene carbonate solution [157]. PEDOT film was oxidized to increase area and roughness. Voltammograms of a mix of AA and UA shows a signal separation of ca. 300 mV. Linear sweep voltammetry was employed for AA determination achieving a LOD of 45 μM. Commercial CNTs were carboxylated by oxidization into a mix of nitric and hydrochloric acid, then the nanotubes were drop-cast on top of glassy carbon electrode from aqueous suspension [158]. This surface was covered with PEDOT by potentiostatic polymerization. Voltammograms of 1 mM AA solution showed a current signal 10-fold larger with final modified surface respect to bare electrode signal, and a separation of 520 mV between UA and AA. Chronoamperometric techniques were used for AA determination reporting a linear range of 1.0 × 10^−4^–2.0 × 10^−2^ M with a LOD of 4.2 μM. Similarly, Sener C. et al. developed a sensor by polymerization of glyoxal-bis(2-hydroxyanil) onto a glassy carbon electrode [159]. Voltammograms with the modified surface show separated signals for AA and UA of ca. 230 mV. A LOD of 0.26 μM was reported using DPV with recoveries of 97.7–101.2%. The selectivity can be attributed to functional oxygen groups of the polymer involved in the electron transfer process.

### 3.4. Glucose

Perhaps the most widely recognized electrochemical sensor is the glucometer [160]. The first electrochemical biosensor was developed by Clark and Lyons in 1962 [161], where Glucose oxidase (GOx) was used as the enzyme to catalyze glucose oxidation at its flavin adenine dinucleotide cofactor FAD. Currently, sensor research is focused on developing non-invasive devices for glucose determination using new materials as conducting polymers, carbon materials, or metallic nanoparticles [162].

#### 3.4.1. Enzymatic Sensors

Thunyakontirakun W. et al. built a biosensor based on electrogenerated poly (pyrrole-3-carboxylic acid) into a composite film with GO and immobilized GOx [163]. A LOD of 50 μM was obtained by amperometric measurements. A novel subcutaneous needle-like device was developed for monitoring glucose [164]. The sensor contains four layers (i) potentiodynamically plated platinum on a gold surface, (ii) electrochemically deposited PANI film, (iii) GOx cross-linked with glutaraldehyde, and (iv) a bi-layer membrane of polyurethane of different thickness. SEM micrographs showed PANI nanofibers with a diameter size between 80 nm and 110 nm and length about 1 mm, interconnected each other to form a network. The sensor Pt/PANI/GOx/PU/E-PU was subcutaneously implanted in male Sprague-Dawley rats showing a lifetime of 26 days. A composite was prepared on a glassy carbon electrode by potential sweeps from an aqueous dispersion containing polyvinylpyrrolidone, polyaniline, and gold nanoparticles [165]. After the electropolymerization, GOx was absorbed on top of the composite, and a Nafion film was dropped onto the surface. The surface denoted as PANI-PVP-AuNP, showed a regular flower-like structure with a rough surface. A LOD of 10 μM was reported for glucose by chronoamperometric measurements. A sensor composed of PANI, graphene, and GOx was prepared for glucose determination [166]. Pt/PANI-GRA/GS-GOx electrodes show PANI rod-like structures with a diameter of about 300 nm and a length of some 1.5 mm. The amperometric technique was employed for glucose determination achieving a LOD of 2.77 μM and linear range of 1.0 × 10^−5^–1.48 × 10^−3^ M. The sensor showed high selectivity and recoveries ranging from 95.6% to 104.9% for glucose determination in plasma samples. Hybrid composite of PANI, montmorillonite (MMT), and palladium nanoparticles was reported by Zheng H., et al. [167]. The composite was electrochemically polymerized from an aqueous dispersion of aniline with MMT by potential sweeps. Then, the surface was decorated with PtNP by electrochemical deposition from H_2_Pt_2_Cl_6_ solutions followed by deposition of a mix of chitosan-GOx. SEM analysis reveals a rod-like PANI structure uniformly distributed on the platinum surface, decorated with MMT and spherical PtNP with an average size of 60 nm. Glucose determination was carried out employing a chronoamperometric method achieving a LOD of 0.1 μM, with a linear range 1.0 × 10^−5^–1.94 × 10^−3^ M and recoveries of 95.1–103.2% into human serum blood samples. A complex biocomposite design for glucose determination was presented by Sun L. et al. [168] based on poly (3-anilineboronic acid) (PAB). Sensors denoted as Au/MWCNT/Pd_plate_/GOx-PAB-PdNP/CS were tested for glucose determination by chronoamperometric methods into aqueous PBS solutions at pH 7.0. A LOD of 0.1 μM with a linear range of 2 × 10^−6^–4.5 × 10^−3^ M, and a repeatability of 7.1% as RSD (*n* = 5) for 5 mM glucose in the presence of 0.5 mM of AA and UA. PEDOT films were electrochemically deposited on a Pt electrode with bovine serum albumin as a platform for Pt/PEDOT-BSA/AuNP-GOx sensors [169]. FTIR, analysis shows no disruption of PEDOT film by the posterior modifications. Glucose determination was carried out with chronoamperometric measurements achieving a linear range of 4.16 × 10^−4^–5.0 × 10^−2^ M with an R^2^ of 0.9967. 1,3-Bis(2-pyridylimino)isoindole palladium complex bearing 3,4-ethylenedioxitiophene (EDOT-PdBPI) was electrochemical co-polymerized with 4-amino-N-(2,5-di(thiophene-2yl)-1H-pyrrol-1-yl)benzamide (HKCN) by potential sweeps onto a graphite rod electrodes [170]. An amperometric technique was employed for glucose determination achieving a LOD of 176 μM. The sensor performance was evaluated with commercial beverages and compared with a spectrophotometric method achieving recoveries of ca. 98%. A film composed of PEDOT, polyacrylic acid, and poly (4-lithium styrenesulphonic acid) PSSLi was electrodeposited on platinum electrode followed by GOx immobilization assisted with a carbodiimide [171]. Glucose determination was carried out into commercial juice and honey samples achieving a LOD of 290 μM with a linear range 9.6 × 10^−4^–3.0 × 10^−3^ M. The same research group reported the synthesis of similar surfaces employing the same basic principles and optimizing the procedure of GOx immobilization [172]. Methylene blue (MB) was electrochemically polymerized onto PAMAN dendrimer/pencil graphite electrode (PGE) followed by glucose dehydrogenase (GHD) immobilization to build a biosensor for flow injection analysis (FIA) [173]. Voltammograms of aqueous glucose solution with NAD+ on PGE/PAMAN/pMB-GHD surface showed an electrocatalytic signal with a reduction of onset potential of ca. 400 mV with respect to the surface without the conducting polymer. A LOD of 4.0 μM, with a linear range of 1.0 mM–1000 μM and recoveries ranging from 96% to 104% were reported into samples of artificial blood serum.

#### 3.4.2. Non-Enzymatic Sensors

A conducting composite of PPy with silver nanoparticles was reported by Poletti et al. for glucose sensing [174]. The pPy-AgNP composite was dispersed into water and then drop-cast on a glassy carbon electrode. SEM and TEM analysis confirmed the formation of spherical silver particles incorporated and distributed in the polymeric matrix. Ag^+^ reduction was accompanied by monomer oxidation which led to Py polymerization. The glucose determination was evaluated chronoamperometrically using artificial human saliva, achieving a LOD of 3.6 μM. PPy was also potentiostatically electrodeposited on a graphite electrode [175]. SEM micrographs revealed polymer wire-like structures covered with nickel nanoflakes. Chronoamperometry was used to determine glucose in alkaline solution, achieving a linear range 1.0 × 10^−6^–4.86 × 10^−3^ M with a LOD of 0.3 μM attributed to the chemical reactivity of the redox pair Ni(OH)_2_/NiOOH, which showed excellent electrocatalysis behavior for glucose. PANI was drop-cast onto a clean glassy carbon surface followed by dipping into a gold nanoparticle dispersion to obtain a GC/PANI/AuNP sensor with a LOD of 100 mM to glucose [176]. A hybrid nanostructure composed of MWCNT, gold nanoparticles, and poly (2-aminothiophenol) (pAT), were deposited on a glassy carbon electrode, for glucose determination [177]. Glucose voltammograms showed a well-defined peak obtained in the reversal sweep because of the gold oxide formation indicating an electrocatalytic effect. The peak current of LSV shows a linear dependence with the glucose concentration from 1.0 × 10^−4^ M to 3.0 × 10^−2^ M achieving a LOD of 3.7 μM. A fluorine-doped tin oxide, (FTO) surface was modified with a nanocomposite of electrodeposited poly (aniline blue) PAnB and gold nanoparticles on FTO/AnB/AuNP [178]. SEM micrographs showed aggregated gold nanoparticles with (111) and (200) crystal planes. Voltammograms of glucose showed oxidation and reduction current peaks in the modified electrodes (not appreciated on non-modified surfaces). Glucose detection was carried out with an amperometric technique achieving a LOD of 0.4 μM, and a linear range of 0 to 50 μM. Synergic effect in the nanomaterial-polymer ensured good contact and high electron transfer rate. A similar composite PANI-nickel nanoparticles was reported for glucose detection [179]. Face-centered cubic nickel oxide was dispersed in the polymer matrix which catalyzed the glucose reaction with respect to the bare electrode increasing with the pH value of the solution. An amperometric determination was tested by successive addition of glucose, reporting a LOD of 0.19 μM. Poly (hydroxymethyl-3,4-ethylendioxythiophene) (PHMeDOT) films were deposited by chronoamperometric techniques on a clean glassy carbon electrode [180]. This surface was used for the glucose determination in real blood samples extracted from different individuals. The results were compared with a commercial “One TouchMini^®^” blood glucose meter, obtaining a relative difference of ca 1.75%. Another sensor based on a composite of PEDOT:PSS with MWCNTs and copper oxide particles has been used for the glucose determination with a linear range up to 10 mM and a LOD of 230 mM by chronoamperometry [181]. A electrochemically nanostructured phenylboronic acid-grafted poly (3,4-ethylenedioxythiophene) (poly (EDOT-PBA)) was developed for glucose determination [182]. SEM micrographs revealed a tubular-type structure while contact angle analysis showed film hydrophilicity. The trials of the absorption of glucose onto the modified surface were carried out with the QCM technique, shown a dependence between the frequencies of crystal with glucose concentrations. A linear range of 0.1 to 50 mM and a LOD of 5.0 μM was achieved with the EIS technique. A nanocomposite of rGO, PEDOT, and nickel nanoparticles was prepared by electrochemical deposition [183]. SEM analysis showed a rough 3D microstructure, due to the GO presence decorated with nickel rod-like structures with an average length of ca 50–100 nm and a diameter of 20 nm. Amperometric measurements were made in NaOH aqueous solution obtaining a LOD of 0.8 μM. Aqueous solutions of 0.1 mM of DA, UA, and AA were used as interferents founding an excellent selectivity to glucose. The selectivity of this sensor was attributed to the nickel nanoparticles. Similarly, a film of PEDOT:PSS decorated with nickel nanostructures was reported by Mazloum-Ardakani et al. [184]. A LOD of 0.69 μM and a linear range of 2.5 to 1100 μM were obtained for glucose in chronoamperometric measurements. A MIP composed of acrylamide and aminophenyl boronic acid on poly(tertiophene), pTBA was developed by Kim et al. [185]. The film was deposited on a screen-printed carbon electrode previously covered with gold nanoparticles (SPCE/AuNP/pTBA-MICP). This sensor was employed for glucose determination into physiological samples like saliva and finger-prick blood. A LOD of 0.19 μM with a linear range of 3.2 × 10^−7^–1.0 × 10^−3^ M was reported using amperometric measurements. Poly (terthiophene) played an important role as a template for boosting the surface area as well as the conductivity. A composite of PEDOT, GO, and copper nanoparticles was electrogenerated for glucose determination [186]. SEM micrographs showed a rough and wrinkled yet uniform microstructure of polymer with well-distributed GO and cubic copper particles of ca. 100 nm. A chronoamperometric technique was employed to measure glucose in real human serum samples reporting a LOD of 47 nM with a linear range of 1.0 × 10^−7^–1.3 × 10^−3^ M and a repeatability of 3.3% as RSD for measuring 0.1 mM glucose with a single modified electrode 10 times. The sensor was also compared with a commercial automatic analyzer achieving an RSD of 4.6%. The good performance of this sensor is attributed to good contact and synergic effect between metallic particles, carbon materials, and conducting polymer. A very similar composite was reported by Wu et al. [187]. This sensor was composed of PEDOT, carbon nanotubes, and copper oxide nanoparticles (GC/PEDOT-CNT-Cu_2_ONP). SEM and TEM micrographs showed well defined six corner star-like microcrystal particles of copper(I) oxide that increase surface area. Amperometric measurements were made by adding successively amounts of glucose. A fast-current response (<4 s) was observed for each glucose addition with a LOD of 40 nM. In addition, the sensor retains 94.9% of its initial current response after 28 days measuring 4 mM glucose. A poly (o-phenylenediamine) (pPD) film covered with silver nanoparticles was employed for the electrochemical determination of glucose [188]. Chronoamperometry was employed for the glucose determination reporting a LOD of 12 μM and recoveries of 94.2–103.4% in human blood samples. Following the same line, Wang et al. reported a similar pPD modified surface with copper nanoparticles resulting in a LOD of 0.25 mM and a linear range of 5.0–1.6 mM for glucose in human blood samples [189].

Glucose sensors based on glucose oxidase usually show high selectivity; however, the scientific community still needs to face important challenges such as the preservation of the surface conductivity and the homogeneous immobilization of the enzyme in the conducting polymer. Carbon materials and metallic nanoparticles in a conducting polymer matrix boost the sensor conductivity resulting in relatively good LODs. On the other hand, the design and construction of orderly-nanostructured enzyme sensors remain an unexplored potential research area aiming to avoid blocking active sites on the electrode surface. Developing enzyme-less sensors for glucose determination is an active field of research. Good results are obtained by preparing nanocomposites of functionalized conducting polymers e.g., with boronic acids. Molecular imprinted polymers are also used as another interesting way for selective glucose determination. Selectivity is enhanced by using metallic nanoparticles where the metal or metal oxides onto the surface play a crucial role in the mechanism of the electronic exchange process.

### 3.5. Hydrogen Peroxide

Hydrogen peroxide, H_2_O_2_, is widely used in many fields such as clinical [190], industrial [191], and environmental analysis [192]; it is also a byproduct of several oxidative biological reactions. It is essential to determine H_2_O_2_ concentration for studying different kinds of disorders in the human body such as cancer, Alzheimer’s, and Parkinson’s, as well as in various industrial applications [193]. Electrochemical sensors based on enzymes were developed as the third-generation of biosensors [194]. Nevertheless, enzyme-free sensors have attracted increasing interest due to their high stability and low-cost [195]

#### 3.5.1. Enzymatic Sensors

Hydrogen peroxide was sensed by a ternary nanocomposite of PEDOT:PSS, reduced graphene oxide and gold nanoparticles assembled with horseradish peroxidase (HRP) on a screen-printed gold electrode (HRP/AuNPs/rGO/PEDOT:PSS/SPGE) [196]. PEDOT:PSS acted as an effective π–π stacking stabilizer for rGO. AuNPs were distributed homogeneously working as nanoscale spacers which allowed access to both faces of PEDOT:PSS and graphene sheets increasing significantly the active surface area of the electrode. Amperometric measurements were used to sensed H_2_O_2_ concentration in a linear range from 5 μM to 400 μM and with a LOD of 80 nM. The repeatability for *n* = 5 showed an RSD of 4.2% for 50 µM H_2_O_2_. A film consisting of poly (γ-glutamic acid) modified with 3-aminothiophene (ATh-γ-PGA) was prepared together with HRP and nafion for the determination of H_2_O_2_ [197]. By cyclic voltammetry, thiophene groups in the ATh-γ-PGA/GE were electropolymerized into acetonitrile solution containing LiClO_4_. Nafion/HRP/ATh-γ-PGA/GE sensors showed two linear ranges for H_2_O_2_ concentrations, from 10 pM to 10 nM and from 10 nM to 10 μM, with a LOD of 3 pM. The sensor showed an RSD of 2.4% for eight assays in 10 nM H_2_O_2_. Chen et al. fabricated a hydrogen peroxide sensor of HRP in an electrogenerated poly (aniline-co-N-methylthionine) film on platinum foil [198]. After enzyme immobilization, smaller nanoparticles appeared increasing the roughness and surface area of the electrode. Copolymer film reduced the background interference from the platinum electrode, acting also as a mediator which formed HRP (Fe^3+^). The PAN-PNMThH film also provided a biocompatible platform for HRP immobilization. Amperometric detection of H_2_O_2_ showed a linear response ranging from 5.0 μM to 60.0 mM H_2_O_2_ with a detection limit of 3.2 μM. A hydrogen peroxide sensor based on PANI together with acrylic acid (AcA), ethylene glycol diglycidyl ether (EGDE), and Soybean seed coat peroxidase (SBP) (SBP/poly (EGDE-AcA-ANI)/GCE) was developed by Torres et al. [199]. PANI enhanced the electron transfer process acting as a good electronic conductor, the acrylate played a role as counter ion achieving electrical neutrality and EGDE worked as a cross-linker. The quantitative analysis of H_2_O_2_ using an amperometric technique showed a linear response in the range of 5.0–50 µM with a LOD of 2.2 µM.

#### 3.5.2. Non-Enzymatic Sensors

A PEDOT film together with rGO was deposited on a GCE for hydrogen peroxide determination [200]. EDOT and GO were mixed in acetonitrile and drop-cast in the GCE, then potential sweeps were applied for EDOT polymerization and GO reduction. A crumpled and rough surface was observed for the PEDOT/rGO film. This morphology produced a larger electrochemically active area which enhanced radical oxygen reduction reaction (ORR) activity. Wang et al. fabricated a selective and long-term stable electrode for H_2_O_2_ determination with Prussian blue (PB) nanoparticles and PEDOT on glassy carbon electrode (PEDOT/PBNPs/GCE) [201]. PEDOT was polymerized around the PB nanoparticles connecting them as a covering shell. As a result, the PEDOT/PBNPs film showed a porous grape-like microstructure. The composite enhanced the electron transfer and conductivity allowing detection of H_2_O_2_ in concentrations ranging from 0.5 μM to 839 μM, with a LOD of 0.16 μM. Following the same line, a PEDOT/PBNPs/Pt sensor was developed by Lete et al. obtaining an amperometric linear response for H_2_O_2_ concentration ranging from 5 μM to 1mM with a LOD of 1.4 μM [202]. Electrogenerated poly (2-aminophenylbenzimidazole) (poly2AB) and gold nanoparticles on pencil graphite electrode (poly2AB/AuNPs/PGE) has also been used as H_2_O_2_ sensor [203]. AuNPs appeared as homogeneous spherical particles that increased the conductivity of the P2AB catalyzing the reduction reaction of H_2_O_2_. Amperometric measurements in PBS solution at pH 6.5 revealed a linear response in the range of 60 µM to 100 mM with a LOD of 36.7 μM of H_2_O_2_. Poly (methylene blue) (PMB), Ag nanocrystals (AgNCs), and graphene carbon spheres (GS) nanocomposite on glassy carbon electrode (Ag/PMB/GS/GCE) were used for hydrogen peroxide detection [204]. GS had a wrinkled texture, showing a rough surface with pores that favor electron transport. The thin PMB layer acted as a mediator adsorbing and binding the Ag^+^ ions. The morphology of AgNCs showed dendritic-like structures. The carbon spheres act as nano-spacers preventing the aggregation of graphene, while poly(MB) benefits the crystals’ growth during the reduction of AgNCs. Ag/PMB/GS/GCE amperometric sensor showed a linear response for H_2_O_2_ concentration ranging from 0.5 μM to 1.11 mM with a LOD of 0.15 μM. The sensor retained 96.5% of the original value after one week of consecutive measurements. A copolymer of poly (pyrrole-3-carboxylic acid) (PPy3C) with polypyrrole on mesoporous platinum (MPrPt) at a boron-doped diamond electrode (BDD) was used for nonenzymatic, free of oxygen interference sensing of hydrogen peroxide [205]. After Pt deposition, mesoporous and snow-flake-like nanoclusters were formed. Electrodeposited copolymer increased the porosity of the structure allowing molecules to permeate through the polymer film. The PPy3C-PPy/MPrPt/BDD electrode showed an amperometric linear response for H_2_O_2_ concentration from 5 μM to 49 mM with a LOD of 2 μM. The enhancement of the current peak was related to the increased conductivity by the copolymer and the electrocatalytic reduction of PtO_2_/PtO.

The ability of the sensors to give an accurate measurement of H_2_O_2_ in the presence of interfering species is fundamental. The interferences usually present in the biomedical samples are DA, UA, KNO_2_, KNO_3_, and AA. Chronoamperometric techniques allow for the possibility of good separation between species because each of them has a different potential at which they reduce or oxidize. Enzymatic sensors display high selectivity towards interfering species ensuring the specific reduction of hydrogen peroxide. However, the stabilization of enzymes might be a hard issue to overcome. Enzymes are easily degraded, thus enzyme-less sensor fabrication may also result in an interesting approach. Researchers tackle the loss of selectivity by the lack of an enzyme using conducting polymers composites with e.g., Prussian blue which acts as a more stable artificial enzyme.

Table 1 enlists a series of electrochemical sensors based on conducting polymers for detection of the various important biomarkers reviewed up to this point. Electrode architecture, name of the conducting polymer, synthetic approach, analyte, detection technique, LOD, and linear range are provided as well as the reference where the information is available.

### 3.6. Diverse Pharmaceuticals

#### 3.6.1. Metronidazole (MNZ)

**Figure 16:**
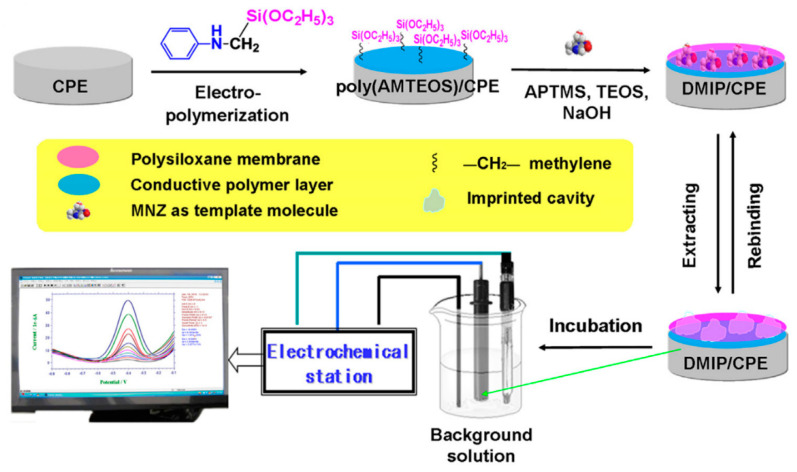
Schematic illustration for the preparation of duplex molecularly imprinted polymer/carbon paste electrodes (DMIP/CPE). Modified with permission from [207]. Copyright 2016 Elsevier.

MNZ is a nitro-compound used for treating diseases caused by protozoa or anaerobic bacteria [206]. This nitro-compound was sensed with a duplex molecularly imprinted polymer (DMIP) hybrid film composed of poly (anilinomethyltriethoxysilane) (poly (AMTEOS)) and an imprinted polysiloxane layer on a carbon paste electrode (DMIP/CPE) [207]. Figure 16 shows a schematic preparation of DMIP/CPE for sensing of MNZ. Poly (AMTEOS) was electrogenerated at the CPE by cyclic voltammetry. Then, a solution containing 3-aminopropyltriethoxysilane (APTMS), tetraethyl orthosilicate (TEOS), 2-ethoxyethanol, and MNZ, was drop-cast onto the modified electrode, where triethoxysilyl groups were hydrolyzed forming a MIP film. DMIP/CPE exhibited a rough surface, because of the formation of imprinted MNZ cavities, which increased the surface area. Electrocatalytic activity and recognition were enhanced due to (i) the amino group from APTMS that forms hydrogen bonds with the oxygen or nitrogen atom in MNZ, and (ii) the phenyl units of the conductive polymer with “π–π stacking” interaction with the aromatic heterocycle of MNZ. Under optimized conditions, the reduction peak currents were linearly proportional to the MNZ concentrations in the range from 0.4 μM to 0.2 mM with a LOD of 91 nM by using a DPV sensor.

**Figure 17:**
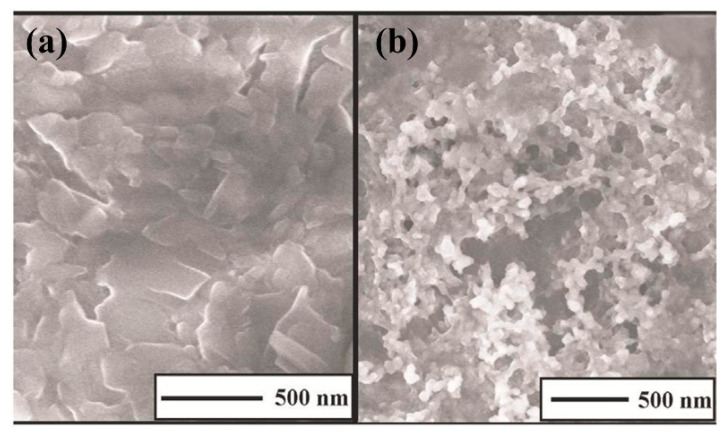
Micrograph of molecularly imprinted polymers/golf nanoparticles/glassy carbon electrodes (MIP/AuNPs/GCE) microstructure (**a**) before and (**b**) after metronidazole extraction. Modified with permission from [208]. Copyright 2015 Elsevier.

Another electrode based on molecularly imprinted polymer was reported with gold nanoparticles for MNZ sensing [208]. A suspension of AuNPs and chitosan was dropped on the surface of the GCE. The modified AuNPs/GCE was immersed in an aqueous solution containing CuSO_4_, H_2_SO_4_, NaCl, MNZ, and melamine, where electrodeposition was carried by cyclic voltammetry. Then, MNZ was extracted in Britton–Robinson buffer with multiple cycles. The MIP/AuNPs/GCE showed folded polymeric layers before MNZ extraction (see Figure 17a). After the template was removed, a rougher morphology was observed (Figure 17b). The polymeric film emulates the microenvironment for the enzymatic reaction, which is better catalyzed in the large surface area of the polymer. Using DPV, the response to metronidazole was linear in the concentration range of 0.5 μM to 1000 μM with a LOD of 0.12 μM.

Similarly, MNZ was determined with a three-dimensional graphene-like carbon architecture (3D-HPG) and polythionine (PTH) modified glassy carbon electrode [209]. The PTH-modified GCE was prepared by cyclic voltammetry in H_2_SO_4_ solution containing thionine. The 3D-HPG suspension was cast on the surface of the modified electrode and dried under an infrared lamp. After the drop-casting, a continuous 3D porous network with macropores was observed. The PTH film increased the electrical conductivity and the 3D-HPG accelerated the electron transfer rate due to its large high surface area, greatly improving the electrochemical sensing with a LOD of 1 nM. The reusability was determined by seven successive measurements at the electrode showing an RSD of 7.3%.

#### 3.6.2. 2-Amino-9-[(2-Hydroxyethoxy) Methyl]-6,9-Dihydro-3H-Purin-6-One (Acyclovir)

**Figure 18:**
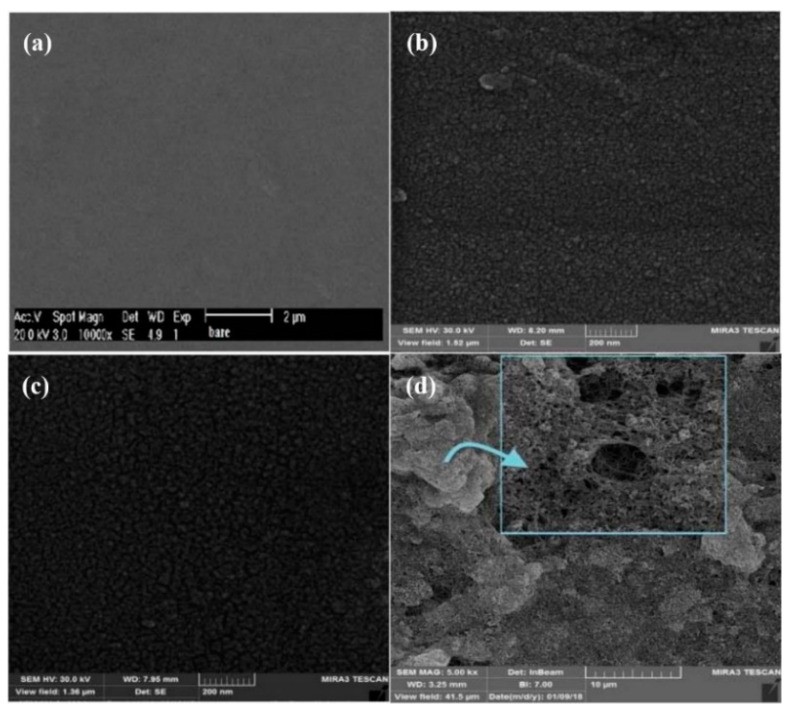
FESEM images of (**a**) bare standard GCE, (**b**) nanoporous GCE, (**c**) PCC/nanoporous GCE and (**d**) CS-MWCNTs+TiO_2_NPs/PCC/nanoporous GCE. Reproduced with permission from [214]. Copyright 2018 Electrochemical Society, Inc.

Acyclovir (ACV) is a synthetic deoxyguanosine analog used as an antiviral drug [210] for the treatment of herpes simplex, herpes zoster infections, primary genital herpes, herpetic encephalitis [211]. Acyclovir triphosphate also prevents DNA synthesis by acting as a chain terminator [212]. A sensitive sensor made of poly-eriochrome black T, (PEBT) deposited on a glassy carbon electrode (PEBT/GCE) was used for detecting Acyclovir by DPV [213]. The PEBT film was potentiodynamically electrogenerated on a GCE surface in NaOH solution. After deposition, uniform branch-like structures were observed enlarging the effective electrode surface approximately 2.4 times. The peak current was linearly related to Acyclovir concentration in an acetate buffer solution with a LOD of 12 nM. An RSD of 2.3% was obtained for ten successive measurements of 0.5 mM ACV. A sensor based on MWCNTs, titanium dioxide nanoparticles (TiO_2_NPs), and electrogenerated poly-catechol (PCC) as a polymeric matrix into a nanoporous GCE was developed for Acyclovir determination [214]. The nanoporous GCE (see Figure 18b) showed a rougher surface compared to the standard GCE (see Figure 18a). Increased porosity was observed after PCC deposition (see Figure 18c) showing randomly tangled wire-like carbon nanotubes and granular morphology of TiO_2_NPs (see Figure 18d). The polymeric film improved the catalytic activity toward the ACV oxidation due to its higher surface area and conductivity. The response of the electrode to the ACV concentration using DPASV was linear from 0.03 μM to 1.0 μM with a LOD of 10 nM. An overoxidized PPy film was electrochemically deposited on carboxylate carbon nanotubes (CNT-CA) modified glassy carbon electrode from an aqueous solution of pyrrole and tiron [215]. Micrographs showed thin PPy film densely covered CNTs as a nanosized backbone improving the surface electron transfer. A tenfold larger current signal was achieved for ACV with CV with respect to the bare GC electrode. This modified electrode was used for the determination of ACV content in tablet and ampule preparations resulting in an acceptable accuracy of 104.9% and a LOD of 10 nM.

#### 3.6.3. Ciprofloxacin (CFX)

**Figure 19:**
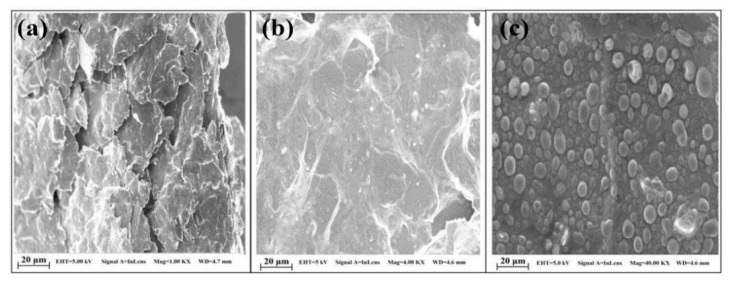
FE-SEM micrographs of (**a**) CPE, (**b**) PEB/CPE and (**c**) SDS/PEB/CPE. Reproduced with permission from [218]. Copyright 2019 Wiley-Blackwell Publishing Ltd.

CFX is a drug widely used in the treatment of numerous bacterial diseases such as pulmonary, respiratory [216], skin, urinary, ocular, and digestive infections [217]. A novel sensor based on anionic surfactant SDS, and polymer-modified on CPE has been developed for CFX sensing [218]. Poly (evans blue) monomer was electrodeposited by cyclic voltammetry on CPE followed by SDS immobilization obtaining SDS/PEB/CPE sensor. The pristine surface contained irregular flake structures from the graphite paste (see Figure 19a). After the polymeric film was added, the surface turned more uniform and regular (see Figure 19b). Whereas at the SDS/PEB/CPE surface, the absorbed surfactant molecules showed globular structures homogeneously distributed on the surface (see Figure 19c). The synergetic effect of poly (Evans blue) and SDS catalyzed the reaction resulting in a LOD of 0.183 μM for CFX. The repeatability was tested by recording the CV outcome for five times at the same electrode giving an RSD of 1.77%. A highly selective electrode based on reduced GO and electrogenerated poly-phenol red (PPR) was designed for CFX detection [219]. The rGO/PPR composite exhibited both characteristics, in which a spongy sheet-like polymer structure was covered by wrinkled rGO nanosheets. The porosity observed in the rGO/PPR film increased the total surface area because of the electrostatic interactions between unoxidized oxygen atoms and the conducting polymer. Under optimal conditions, current response showed a linear relationship for CFX concentration ranging from 50 nM to 400 µM with a low LOD of 2 nM.

#### 3.6.4. 17-β-Estradiol (E2)

**Figure 20:**
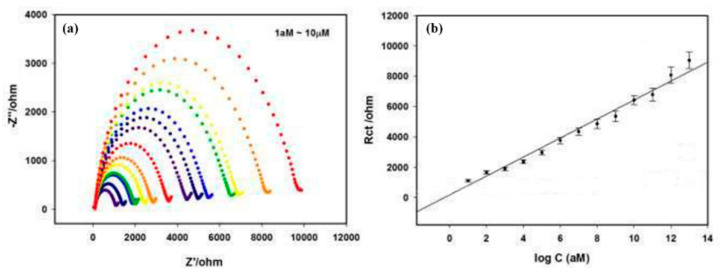
(**a**) EIS response of the MIP sensor towards 17-β-estradiol in the concentration of 1 aM to 10 μM. (**b**) Calibration curve of the R_ct_ values versus the logarithm concentration of 17-β-estradiol. Modified with permission from [223]. Copyright 2018 Elsevier.

E2 and other natural and synthetic compounds can mimic endogenous hormones [220]. They can interfere with the proper functioning of the hormonal, immune, and nervous systems of mammals [221]. Zhang et al. fabricated a polymeric/enzymatic biosensor for sensing E2 with electrodeposited poly (4,7-bis(5-(3,4-ethylenedioxythiophene)thiophen-2-yl)benzothiadiazole) (Pol) and HRP onto platinum electrode [222]. The Pol/Pt surface showed uniformly distributed granular structures. The formed pores allowed the enzyme to anchor and maintain its catalytic activity. The sensor was effective in the E2 concentration range from 0.1 μM to 200 μM with a LOD of 105 nM. The polymer improved the reaction in two ways (i) acting as an electron mediator increasing the electron transfer between the enzyme’s active center and the electrode surface, and (ii) creating an appropriate microenvironment to immobilize the protein. Poly (3,6-diamino-9-ethylcarbazole) based molecularly imprinted polymer electrode developed by Liu et al. showed to be an ultra-sensitive and selective sensor for detecting 17-β-estradiol even at attomolar concentrations (aM, 10^−18^ M) [223]. The electrodeposition of the monomer (3,6-diamino-9-ethylcarbazole) was carried out by cyclic voltammetry in a mixed solvent solution of ethanol and acetate buffer containing the template (17-β-estradiol). Removal of the template was made by washing in a stirred solution of H_2_SO_4_/ethanol. The surface was observed to be rough with micro islands of poly (3, 6-diamino-9-ethylcarbazole) working as active sites for the recognition of E2. A linear relationship was found between the R_ct_ value of the EIS response and the logarithm of E2 concentrations (see Figure 20a). The quantification of E2 showed a wide linear range from 1 aM to 10 μM (see Figure 20b) with an exceptionally low LOD of 0.36 aM. The repeatability of the MIP sensor in 100 aM E2 for 5 times gave an RSD of 6.96%.

#### 3.6.5. Paracetamol (PR)

**Figure 21:**
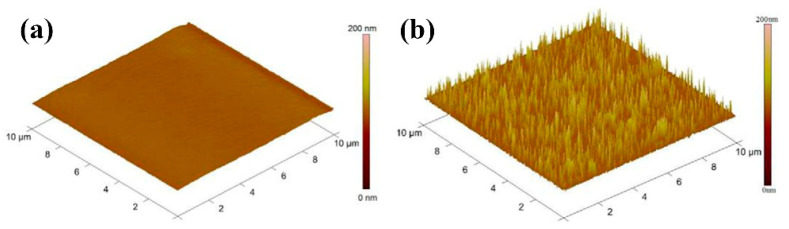
AFM images of the (**a**) unmodified glassy carbon electrode and (**b**) poly Nile blue modified glassy carbon electrode. Modified with permission from [231]. Copyright 2016 Elsevier.

PR or acetaminophen (N-acetyl-p-aminophenol) is an effective analgesic and antipyretic agent is one of the most commonly used medications worldwide [224,225]. Normally, is a safe analgesic agent, but excessive and long-term usage may lead to the accumulation of toxic metabolites, which leads to liver and kidney damage [226]. Electrochemical methods for PR determination are selective, rapid, low cost, and easy in handling in contrast with spectrometry, chromatography, or mass methods [227]. Different dyes were electrodeposited over carbon electrode surfaces as conductive polymeric films for the fabrication of PR electrochemical sensors. A glassy carbon electrode modified with Prussian blue (PB) and a molecularly imprinted polypyrrole was developed for the sensitive determination of PR [228]. PB film was electrodeposited by cyclic voltammetry in an HCl solution containing FeCl_3_·6H_2_O, K_3_[Fe(CN)_6_], and KCl. The activation of the film was carried out in the same solution without the iron species. PB/GCE was immersed in a solution with pyrrole and PR for potentiodynamically deposition onto the surface. PR was extracted in PBS containing KCl. PB film showed irregularly shaped crystals suited in nanoclusters. Using DPV measurements, it was found that the addition of PR not only increased the peak current of PR oxidation but also decreased the current for the PB signal due to partial blocking of the channels. A linear relation was found between the ratio of the currents and the PR concentration in the range of 1.0 nM–0.1 mM with a LOD of 0.53 nM for PR. A voltammetric study of PR was developed using an electrogenerated poly (rhodamine B)-modified CPE [229]. The modified electrode showed a good selectivity and sensitivity with a CV linear response for PR concentration ranging from 20 μM to 90 μM and a LOD of 2.2 μM. Poly (rhodamine B) accelerates the electrochemical reaction and reduces the overpotential which improves the oxidation current signal. Kuskur et al. deposited naphthol green B on the bare carbon paste electrode (CPE) surface by cyclic voltammetry [230]. When naphthol green B was electropolymerized the roughness of the surface in the graphite flakes increased which was reflected in the higher peak current response. By CV, the modified electrode displayed sensitivity, selectivity, and stability for the determination of PR with a LOD of 1.6 μM. Chitravathi and Munichandraiah modified a GCE with a polymeric film of Nile blue using CV [231]. The formation of PNB on the surface was confirmed with the observation of a rough surface, compared to the unmodified electrode (see Figure 21) showing an increased surface area which improved the sensitivity in voltammetric determinations. EIS revealed values of R_ct_ for bare GCE and PNB/GCE of 3200 kΩ and 980 kΩ, respectively, implying that the polymeric film increased the conductivity of the sensor. A wide linear range was observed for PR sensing from 0.2 μM to 16.2 μM with a LOD of 80 nM and an RSD of 1.4% calculated for five measurements into a mixture of 0.1 mM of PR, tramadol, and caffeine.

**Figure 22:**
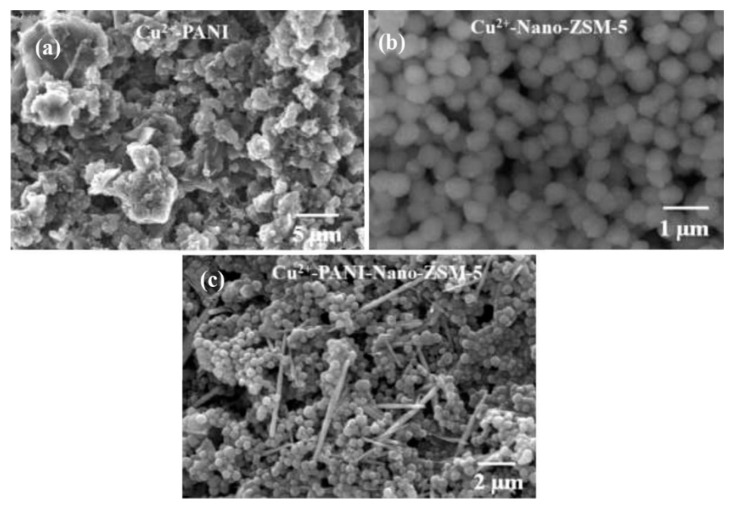
SEM images of (**a**) Cu^2+^-PANI, (**b**) Cu^2+^-Nano-ZSM-5, and (**c**) Cu^2+^-PANI-Nano-ZSM-5 nanocomposite. Reproduced with permission from [237]. Copyright 2015 Elsevier.

Li et al. fabricated a electrogenerated poly (3-Methylthiophene) (P3MT)/rGO modified GCE for PR sensing [232]. P3MT/RGO/GCE sensor displayed a rough wrinkled surface indicating that (i) rGO was dispersed uniformly, and (ii) a homogeneous deposition of P3MT was achieved. Under optimal conditions, the anodic peak current changed linearly with PR concentration with a LOD of 25 nM. P3MT/RGO/GCE sensor not only increased reversibility of the redox reaction but also enhanced the response, which was proof of a remarkable synergistic effect between polymer and carbon material. Electrodeposited poly luminol (PLum)/f-MWCNTs modified GCE was developed as a highly sensitive PR sensor [233]. The bare GC electrode showed a non-porous uniform surface, while PLum/f-MWCNTs/GCE displayed a tube-shaped structure. Using SWASV in BR buffer solution at pH 7.0, the electrode showed two linear responses in the range of 40 nM to 32.2 μM and 32.2 μM to 172.2 μM with a LOD of 25 nM. Electrodes based on PEDOT, poly (4-lithium styrenesulfonic acid) (PSSLi), and MWCNT were electrochemically fabricated for PR sensing [234]. PEDOT:PSSLi:MWCNT displayed a rough, uniform, dense, and compact structure. Carbon nanotubes formed tubular-shape structures which increased the surface area. PEDOT film acted as conductive phase and redox mediator, whereas PSSLi doped with its anion groups the oxidized form of PEDOT improving the mechanical properties of the composite. PEDOT:PSSLi:MWCNT/GCE sensor showed a linear response in the range from 1.5 μM to 500 μM, each concentration in the calibration curve was measured five times, obtaining an RSD of 8.69%. The limits of detection were 80 nM by AdSDPV. PR was detected by a microbial biosensor based on PANI/multiwalled carbon nanotubes on gold electrodes [235]. Lyophilized *Bacillus sp*. cells in PBS solution at pH 7.0 were dip-coated onto the modified MWCNT/PANI/Au electrode followed by immersion in glutaraldehyde solution. The anodic peak current increased dramatically, due to the enzymatic reduction reaction to PR. Amperometric responses showed a LOD of 2.9 µM. The composite enhanced the sensitivity of Au electrode and π-π stacking interactions between MWCNT and PANI provided good stability and conductibility. Kaur and Srivastava developed transition metal ion-exchanged polyaniline-zeolite organic–inorganic hybrid materials (PANI-Nano-ZSM-5) for simultaneous determination of EP, paracetamol, and folic acid [236]. Among all materials, Cu^2+^-PANI-Nano-ZSM-5 modified GCE exhibited the highest electro-catalytic activity. SEM images showed that Cu^2+^-PANI (see Figure 22a) displayed an irregular aggregated morphology. Spherical particles were observed in the case of Cu^2+^-Nano-ZSM-5 (see Figure 22b). After PANI was added, nanorods were produced on the Cu^2+^-Nano-ZSM-5 nanocomposite (see Figure 22c). Cu^2+^-PANI-Nano-ZSM-5/GCE exhibited well-defined anodic and cathodic peaks for PR, showing higher current response than the bare electrode. Under optimal conditions, a wide linear range was obtained from 15 nM to 800 μM with a detection limit of 8 nM for PR [237].

#### 3.6.6. Other Drugs

Acetylsalicylic acid (ASA) is an important nonsteroidal anti-inflammatory [238], analgesic [239] and antipyretic drug [240]. However, is also known to have effects such as gastric acid secretion and dieresis if abused. A new electrochemical sensor made of manganese dioxide (MnO_2_)- antimony trioxide (Sb_2_O_3_) together with PANI on tin oxide (FTO) electrode (MnO_2_-Sb_2_O_3_/PANI//FTO) was used for sensing ASA in urine [241]. The PANI film showed large lumpy shapes and clews structures. After MnO_2_-Sb_2_O_3_ deposition, the composite was agglomerated into a globular structure. Improvement in electron transfer kinetics was attributed to large surface area and high electrocatalytic activity of PANI, MnO_2,_ and Sb_2_O_3_. Under optimal conditions, the sensor showed a LOD of 0.20 nM by DPV. Successive determinations of ASA at 30 nM were performed 30 times in a row resulting in an RSD of 1.28%. An electrochemical imprinted sensor based on PPy, sol-gel, and SiO_2_@Au core-shell nanoparticles showed a linear response to ASA concentration using SWV [242]. The deposition onto a gold electrode was carried out by one step using CV in a solution containing phenyltriethoxysilane, tetraethoxysilane, ethanol, trifluoroacetic acid, ASA, lithium perchlorate, pyrrole, and SiO_2_@AuNPs. Pyrrole increased the stability of the resultant MIP and enhanced the conductivity resulting in a sensor with a LOD of 0.2 nM for ASA. The imprinted sensor was reused for 10 repeated analyses with an RSD of 3.0%.

2-Aminoethanesulphonic acid (Taurine) is an organic acid present in most living organisms. It is one of the most abundant of the low-molecular weight organic compound, e.g., a 70 kg human contains up to 70 g of taurine [243]. Taurine plays an important role in numerous physiological and pharmacological processes such as membrane stabilization, intracellular Ca^2+^ regulation, neuro mediators, and neuromodulator [244]. MICP films have been developed for taurine determination [245]. The platform was made onto GCE, applying several scans of potential in a solution of EDOT, riboflavin-5′-phosphate (FMN), 3-thiophene acetic acid (AAT), and taurine. Taurine was extracted from MICP using a mix of methanol-water 2:5 resulting in a porous cauliflower-like structure. A linear response toward protonated taurine was observed with a slope of 53.8 mV per decade.

(+)-(S)-(6-methoxynaphthalen-2yl)propionic acid (Naproxen or NAP) is a widely non-steroidal anti-inflammatory agent advocated for use in rheumatoid arthritis, degenerative joint disease, and ankylosing spondylitis [246]. MICP of overoxidized PPy on quartz crystal microbalance (QCM) has been reported by Eslami and Alizadeh for NAP sensing [247]. PPy was electrodeposited from an aqueous solution of NaOH, pyrrole, and NAP. Optimized overoxidized sensors were employed for NAP determination achieving a linear dependency in the range of 0.1 µM to 150 µM and a LOD of 40 nM.

3,4-Methylenedioxymethamphetamine (MDMA), commonly known as ecstasy, is a psychoactive recreational drug important whose analytical determination in a wide variety of matrices is a relevant subject in health and forensics sciences [248]. Molecular imprinted film of poly (o-phenylenediamine) (poly (o-PD)) film was electrodeposited on screen-printed carbon electrodes for MDMA determination [249]. After the modified electrode surface was incubated into MDMA solutions for 10 min, a LOD of 0.79 μM was found by SWV. Poly (o-PD) sensors were used for MDMA determination in human blood serum and urine samples with a recovery of 81–91%.

### 3.7. Hydrazine

Hydrazine, N_2_H_4_, and its derivatives have several application fields such as rocket fuel, insecticides, herbicides emulsifiers, textile dyes, and corrosion inhibitors [250]. An inconvenient for the electrochemical sensing is that over conventional surfaces, hydrazine needs a high overpotential for oxidation [251]. For this reason, modified electrodes to reduce the oxidation overpotential is a research challenge. Lignosulfonates (LS) are amorphous aromatic biopolymers, co-produced in the paper industry, which exhibit electrocatalytic behavior towards nicotinamide adenine dinucleotide (NADH) possibly due to the formation of quinone–hydroquinone redox couple [252].

**Figure 23:**
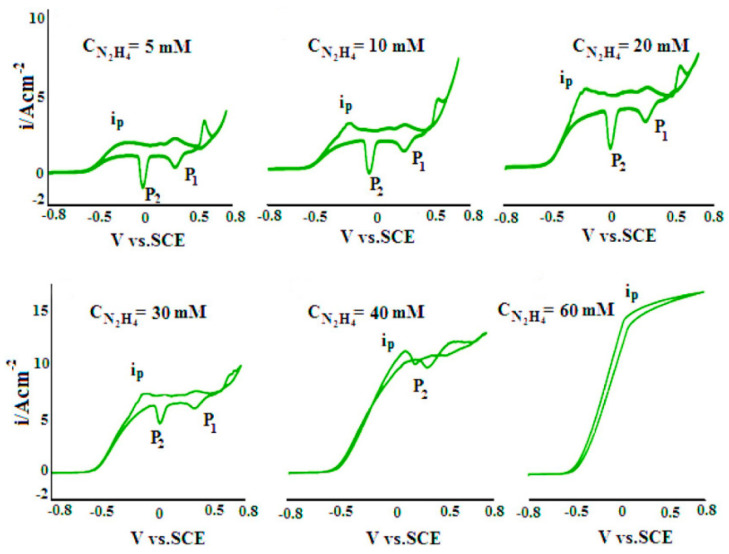
CV responses of the FTO/PANI-gC_3_N_4_/AgNP electrode at a scan rate of 50 mV in the presence of different hydrazine concentrations. Modified with permission from [254]. Copyright 2018 Elsevier.

Rębiś et al. developed a sensor for N_2_H_4_ based on PPy and lignosulfonates [253]. The biocomposite was deposited onto a GCE from an acetonitrile–water solution with Py, LiClO_4,_ and LS. Voltammograms made with GCE/PPy-LS exhibits a catalytic signal activity towards hydrazine. An amperometric method for N_2_H_4_ was developed in aqueous solutions obtaining a LOD of 1.6 μM. The electrocatalytic effect was ascribed to the quinone groups from LS acting as redox mediator and the semiconductivity of the polypyrrole. A carbon nitride, gC_3_N_4_, PANI, and silver nanoparticles composite was tested for N_2_H_4_ sensing [254]. Bulk gC_3_N_4_ was synthesized from melamine precursors, exfoliated, and co-deposited with PANI onto fluorine-doped tin oxide (FTO). FE-SEM shows a net-like structure with good distributed electrogenerated AgNPs. Voltammograms of hydrazine shows a catalytic current dependence with the concentration of hydrazine (see Figure 23). A chronoamperometric method was used for hydrazine determination achieving a LOD of 300 μM.

Copper nanoparticles-decorated polyaniline-derived mesoporous carbon on CGE was used as a sensor for N_2_H_4_ [255]. Mesoporous N-doped carbon nanoparticles (CNP) were synthesized by chemical polymerization of aniline employing silica nanoparticles as a template followed by pyrolysis at 1000 °C. CNP/Cu material has a nanopore structure with an average pore size of 11 nm with homogeneously distributed copper (111) nanoparticles. Hydrazine oxidation showed electrocatalytic activity for CNP/Cu compared to CNP and bare GCE. Nickel-iron nanoparticles (NiFe_2_O_4_NP) were covered with poly (rhodamine) (PRd) by oxidative polymerization were prepared by Lashkenari et al. [256]. PRd@NiFe_2_O_4_ micrographs showed cubic particles with an average size of 80 nm. Voltammograms of hydrazine showed a reduction of the onset potential of ca 200 mV and a larger current of approximately 20% in respect of non-modified surfaces. Ramaraj et al. developed a disposable N_2_H_4_ sensor based on copper(II) hexacyanoferrate complex nanocubes stabilized with poly (diallydimethylamonium chloride) (PDDA) [257]. Screen-printed carbon electrodes were modified by drop-casting the nanoparticles into ethanol. Voltammograms of hydrazine showed a ten-fold increase of current signal with respect to the bare electrode. Hydrazine determination was carried out with a chronoamperometric technique achieving a LOD of 10 nM, a linear range of 30 nM to 570 μM, and recoveries of 96.0–99.1% into real water samples. Besides, 4.9% of the initial current response was lost after continuously measuring a solution of 50 mM hydrazine for 5000 s. A paper-based inkjet-printed sensor for hydrazine determination was developed by Beduk et al. [258]. Three layers of commercial PEDOT:PSS was printed on paper followed by two layers of ZnO precursor ink. After drying on a hot plate at 150 °C, one final layer of nafion was printed resulting in a hydrazine sensor with a LOD of 5 μM by chronoamperometry. Following the same principle, a zinc oxide nanocomposite covered with polythiophene (PTy@ZnO) has been prepared by sol-gel methodology then deposited on GCE for the hydrazine determination [259]. EIS experiments were made with hydrazine founding a notable decrease in electron transfer resistance, indicating a fast electron transfer, in contrast to the bare electrode. An amperometric method was developed for hydrazine determination reporting a LOD of 0.207 μM with a response time below 5 s. A three-dimensional macroporous PEDOT decorated with copper nanoparticles was electrochemically fabricated by Xu et al. [260]. GCE/3D-PEDOT/Cu_x_O exhibits a 3D structure with bumps and open macropores formed by nanofibers and decorated with spherical copper oxide particles of ca. 230 nm size. Hydrazine was detected by amperometric measurements into aqueous solutions at pH 8.0, obtaining a LOD of 0.2 μM and recoveries ranging from 98% to 104% into tap and lake water real samples. Alizarin red S, 1,2-dihydroxy-9,10-anthraquinone-sulfonate (ALS) was potentiodynamically electropolymerized onto pencil graphite electrode obtaining flakes-like structures [261]. The GCE/PALS was employed for hydrazine determination with amperometric methods achieving a LOD of 0.28 μM, a linear range from 1 to 600 mM, and recoveries of 95–108% in water samples. Polydopamine (PDA)-rGO was employed for build an hydrazine sensor achieving a LOD of 10 nM in aqueous solution at pH 7.0 by SWV [262]. This sensor showed a reproducibility ranging from 1% to 4% expressed as RSD.

### 3.8. Nitrites

Nitrites are important in the nitrogen cycle and food preservation, as well as a fertilizing agent [263,264]. However, when ingested, it causes the oxidation of hemoglobin into methemoglobin in the blood, avoiding this protein to bind with oxygen molecule [265]. Nitrite can react with degradation products of meat forming nitrosamines which are carcinogen compounds [266]. The World Health Organization (WHO) reported that nitrite levels in water should stay below 3mg/L (65 μM) [267]. Thus, a precise determination of nitrite is of high importance for the environment and human health.

PEDOT and carbon quantum dots (CQDs) has been used for nitrite sensing by modifying a glassy carbon electrode (CQDs/PEDOT/GCE) [268]. Direct electrochemical polymerization of the composite was performed by potentiostatic methods in an aqueous solution containing CQDs and EDOT. The CQD-PEDOT film was rough and lumpy with small pores throughout the film, enlarging the surface area. The nanocomposite act as a promoter to enhance the kinetics of the electrochemical oxidation process which effectively electro-catalyzes oxidation of nitrite. The CQDs/PEDOT/GCE showed a nitrite linear response with a range from 0.5 μM to 1110 μM and a LOD of 88 nM. A similar approach was followed by Wang et al. by modifying a CGE with PEDOT doped with nano-sized hydroxyapatite (nHAp/PEDOT/GCE) [269]. The electrodeposition was carried out under a potentiostatic regime in a solution containing nHAp and EDOT. The nHAp/PEDOT film exhibited a rough three-dimensional reticular structure. EIS showed a lower R_ct_ for nHAp/PEDOT/GCE if compared to the one obtained in bare GCE, attributed to the large surface area and enhanced conductivity of the nanocomposite. Linear amperometric response for nitrite concentrations ranged from 0.25 μM to 1.05 mM with a LOD of 83 nM. A multilayered film of electrogenerated poly (3,4-ethylenedioxythiophene)/poly (thiomethyl 3,4-ethylenedioxythiophene)/gold nanoparticle (PEDOT-SH/PEDOT/Au) nanocomposite was fabricated by Ge et al. [270]. PEDOT film exhibited nanofiber structures that formed a porous network. PEDOT-SH thickened the nanofibers and formed block structures. AuNPs were distributed uniformly on the porous network directed by bonding interactions with thiol groups. Au/PEDOT-SH/PEDOT/GCE sensor exhibited a LOD of 51 nM and two amperometric linear ranges from 0.15 mM to 1mM and from 1 mM to 16 mM, for nitrite concentration. Zuo et al. designed a sensitive and selective nitrite sensor based on phosphovanadomolybdates H_6_[PMo_9_V_3_O_40_], poly (ethylenimine), poly (3,4-ethylenedioxy thiophene) and gold nanoparticles on glassy carbon (AuNP/PEDOT/PMo_9_V_3_/PEI/GCE) [271]. After GCE modification, a rough surface was obtained showing uniform distribution of gold nanoparticles in the polymer film. This amperometric sensor showed a linear range and LOD of 2.5 nM–1.43 mM and 1.0 nM, respectively. These outstanding results might be related to PEDOT π-conjugation and the presence of sulfur atoms, which chemically bond with the well-distributed gold nanoparticles, improving the electrical conductivity and electron transfer, while polyoxometalates act as a proton and electron reservoir in the electrocatalytic process. Poly (1,5-diaminonaphthalene) together with palladium nanoparticles and MWCNTs on glassy carbon electrode (PdNPs-poly (1,5-DAN)/MWCNTs/GCE) exhibited a high analytical performance for nitrite detection [272]. An amperometric sensor for nitrite showed a peak current proportional to its concentration in a linear range of 0.25 µM to 0.1 mM, with a LOD of 80 nM. Nitrite was determined, as well, using a GCE modified with GO, PPy, and cobalt nanostructures (CoNS/GO/PPy/GCE) [273]. The GO/PPy nanocomposite showed a porous two-dimensional nanoflake structure. Cobalt NS revealed a flower-like crystal structure with open-nanoporous that significantly increase the active surface area ensuring unhindered diffusion of ions and redox substances. Under optimum conditions, nitrite concentration by amperometry showed two different linear ranges, from 1.0 μM to 3.2mM and 6.8 mM to 12mM with a LOD of 15 nM and a response time of 1 s.

### 3.9. Phenolic Compounds

Phenolic compounds are important chemicals used in several industries such as synthetic resins, plants, paints, textile, plastic, pharmaceutical, petroleum, and mine discharges [274,275,276]. However, they are considered a major class of pollutants due to their high toxicity, carcinogenicity, and low biodegradability [277]. According to Khan et al. the WHO has determined 1 μg/L (11 nM for phenol) as the maximum concentration allowed in drinking water [278]. These compounds can cause severe diseases e.g., methemoglobinemia [279], drowsiness [280], and nausea [281]. Therefore, methods for the determination of these compounds in long-term and real-time are of great significance [282,283].

**Figure 24:**
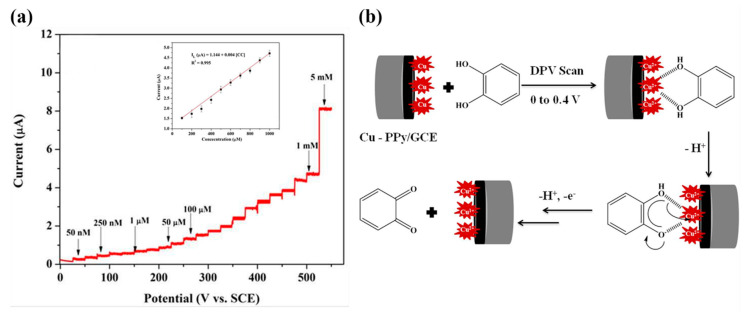
(**a**) Chronoamperogram for the sequential addition of catechol at Cu-PPy/GCE in 0.1 M PBS (pH = 7.0) at 0.3 V vs. SCE. (**b**) Schematic representation of the formation of a five-membered ring with Cu(II) and catechol and further oxidation of catechol at Cu-PPy/GCE. Modified with permission from [287]. Copyright 2017 Electrochemical Society.

Voltammetric determination of (2R,3S)-2-(3,4-dihydroxyphenyl)-3,4-dihydro-2H-chromene-3,5,7-triol (catechin) was performed using a glassy carbon electrode doped with poly (hydroxymethylated-3,4-ethylenedioxythiophene) (PEDOTM) and carboxylic group functionalized single-walled carbon nanotubes (f-SWCNTs) [284]. After potentiostatic deposition of PEDOTM on GCE, f-SWCNTs were drop-cast and dried at room temperature. The nodular and highly porous morphology of the *f*-SWCNTs/PEDOTM/GCE provided a large electroactive area which showed a linear behavior for catechin concentration, in PBS solution at pH 7.00, ranging from 39 nM to 40.84 μM with a LOD of 13 nM. The f-SWCNTs/PEDOTM film highly increased the active surface area for the adsorption of catechin accelerating the electron transfer between electrode and solution, which boosted the current response and improved the sensitivity. Ten successive measurements allowed to determine an RSD of 1.19%. One-dimensional electrogenerated PEDOT-graphene composite (PEDOT-Gr) was used for the detection of resorcinol (RC), hydroquinone (HQ), and catechol (CC) [285]. The graphene sheets showed atomic defects covering the edges and basal planes which produced high nucleation density allowing the formation of PEDOT structures with a 1D morphology in the edges of the sheets. 1D PEDOT-Gr/Ta sensor showed well-defined peaks at 12 mV (HQ), 120 mV (CC), and 512 mV (RC) vs. Ag/AgCl in aqueous solution with linear ranges of 5–250 μM, 0.4–350 μM and 6–2000 μM, and LODs of 60 nM, 80 nM, and 0.16 μM, respectively. The high electron affinity and aromaticity of thiophene with the high conductivity of graphene nanosheets and the specific electron transfer properties accessible only to 1D material all together showed exceptional ability to adsorb and capture electrons of multiple analytes and discriminate between them. Sensitive and selective sensor for CC and HQ was fabricated by Kuskur et al. employing electrodeposited poly (Naphthol green B) modified carbon paste electrode (poly (NGB)/CPE) [286]. The morphology changed from irregularly shaped micrometer-sized flakes of graphite to a uniform arrangement of poly (NGB) molecules on the surface of the electrode. The bare electrode showed amalgamated and indistinguishable signals for CC and HQ, while poly (NG B)/CPE showed a separation of 125 mV. Under optimal conditions, the modified CPE can detect CC and HQ with LODs of 0.19 μM and 0.20 μM, respectively. Improved detection for modified CPE raises due to the formation of stable redox-active layers and high electron transfer efficiency of poly (NGB) acting as mediators for electrocatalysis of biological compounds. A selective non-enzymatic sensor for CC determination was developed using copper-polypyrrole modified GC electrode (Cu-PPy/GCE) [287]. SEM images revealed globular PPy structures resulting in a microporous morphology, while Cu deposits showed pinecone–like morphologies on the micropores of the PPy surface. Under optimized conditions, amperometric measurements showed a linear range from 50 nM to 1.0 mM with a LOD of 10 nM (see Figure 24a). The high electrocatalytic behavior and selectivity of Cu-PPy/GCE towards CC were attributed to the formation of a five-member Cu(II)-o-quinolate intermediate complex and the CC oxidation to o-quinone through the reduction of Cu (II) to Cu (I) that enhances the electron transference (Figure 24b). An RSD of 6.3% was obtained from the current responses of ten successive measurements.

### 3.10. Nitroaromatic Compounds

Nitroaromatic compounds are considered pollutants that commonly infiltrate soil and groundwater because of their usage in industries of rubber, dye, pharma, detergent, resin, paper, and widely in the armament industry as explosives [288,289]. On humans, the toxicological impact is high because they persist for long periods, producing several health problems such as skin damage and necrosis [290]. These kinds of compounds are chemically stable and poorly biodegradable because of their nitro-substituted aromatic structure [291]. However, one advantage for the application of electrochemical methods is the easily reducible nitro groups allowing development sensors with good sensitivity, selectivity, and fast response [292]. Furthermore, modifying electrodes with polymers and metallic nanoparticles had proven to result in a great increase in the recognition of analytes and stability of the electrodes [293].

**Figure 25:**
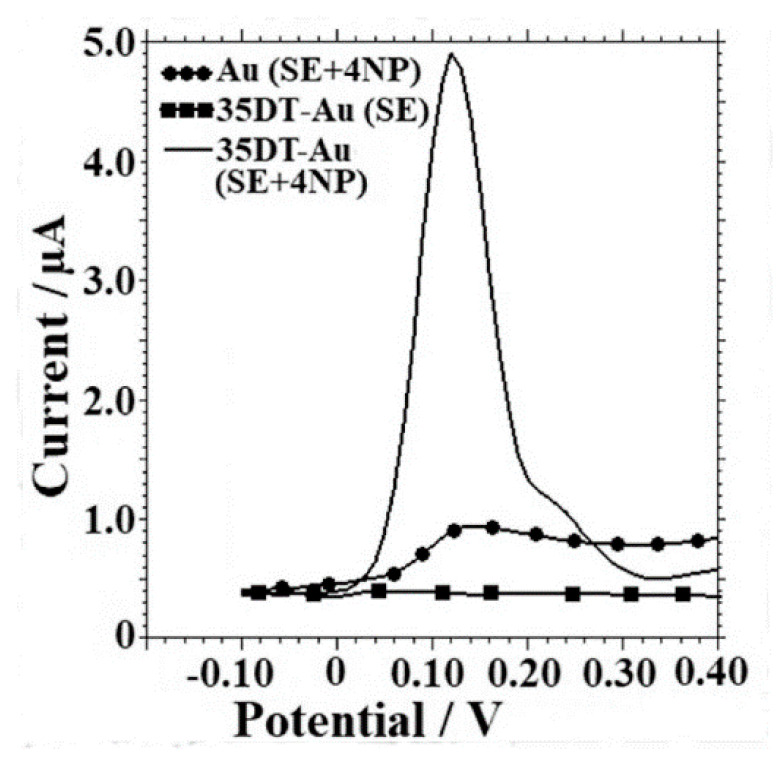
Differential pulse voltammetry of poly (35DT)/Au electrode without 4-nitrophenol (solid square line), a bare gold electrode with 50 mM of 4-nitrophenol (solid circle line), and poly (35DT)/Au electrode with 50 μM of 4-nitrophenol (solid line). Modified with permission from [298]. Copyright 2019 Wiley-VCH Verlag GmbbH and Co.

Nitroaromatic explosive materials such 2,4,6-trinitrotoluene (TNT), 2,4-dinitrotoluene (DNT) and 2,4,6-trinitrophenylmethylnitramine (tetryl) were detected using glassy carbon electrode coated with electrogenerated poly (o-phenylenediamine–aniline) and gold nanoparticles (AuNp/P(o-PDA-co-ANI)/GCE) [294]. Poly (o-PDA) film was able to catalyze nitroaromatic compounds; however, the coating peeled off the surface after several measurements. In the case of PANI film, it exhibited stability through measurements, but it was non-reactive to nitroaromatic compounds. P[o-PDA-co-ANI] exhibited both stability and electroactivity, in which nitroaromatic compounds were detected through π-acceptor/donor interactions. AuNPs provided increased binding because s-/π-donor amine/aniline groups could link gold nanoparticles interacting with the electron-poor nitroaromatic compounds. Linear responses were observed for TNT ranging from 11 µM to 176 µM with a LOD of 9.2 µM, for DNT ranging from 11 µM to 220 µM with a LOD of 7.0 µM, and for tetryl ranging from 17.4 µM to 348.3 µM with a LOD of 13.2 µM. Potentiodynamic deposition of polyalizarin red (PAR) on glassy carbon electrode (PAR/GCE) was proposed by Chen et al. for detection of nitrofurazone, nitrofurantoin, and furazolidone [295]. The reduction peak current of nitrofurazone using PAR/GCE, in HAc-NaAc buffer solution at pH 5.0, was 2.47 times higher than the bare GCE signal. DPV sensor showed a peak current proportional to the concentration in the range of 3.0–50.0 μM and 50.0–200.0 μM, with a LOD of 0.33 μM for nitrofurazone. Analysis of furantoin and furazolidone showed linear ranges of 10–40 μM and 50–140 μM, respectively, with a corresponding LODs of 0.73 μM and 1.56 μM. The repeatability test was performed 15 times where the peak current of the reduction was 94.73% of the initial value. Nitrophenol isomers were sensed using graphite electrodes coated with poly (p-aminobenzene sulfonic acid) (poly (p-ABSA)) film [296]. An electrochemical potentiodynamic deposition was carried out using an aqueous solution containing p-ABSA. o-NP, m-NP, and p-NP isomers were simultaneously determined at 0.119 V, −0.125 V, and 0.027 V vs. SCE using a semi-derivative technique which improved the resolution and enhanced the sensitivity of CV curves. Liner ranges for the oxidation peaks of the intermediate products of nitrophenol were 3–800 mM for o-NP, and 3–700 mM for both m-NP and p-NP. Sensitivity and LODs for o-NP (0.28 mM), m-NP (0.5 mM), and p-NP (0.3 mM) were attributed to the favorable electrocatalytic activity of poly(p-ABSA) towards the oxidation of hydroxyl aminophenol. Nitrofurantoin (NFT), nitrofurazone (NFZ), furaltadone (FTD), and furazolidone (FZD) were successfully determined using a screen-printed carbon electrode coated with overoxidized multi-walled carbon nanotubes and poly (melamine) (PME/MWCNT*/SPCE) [297]. Multifunctional melamine was electropolymerized from an HCl solution by cyclic voltammetry on MWCNT modified electrodes leading to the formation of microporous structures. While the bare SPCE is very hydrophobic with a water contact angle of 136°, the PME/MWCNT*/SPCE displayed a reduced hydrophobic nature with a value of 52.1°. The linear relation from peak current and concentration for NFT, FZD, and NFZ ranged for all from 50 nM to 2.0 μM with LODs of 12 nM, 7 nM, and 6 nM, respectively. The linear response for FTD was higher going from 50 nM to 5.0 μM and a LOD of 14 nM. A gold electrode modified with electrogenerated 3,5-diamino-1,2,4-triazole (35DT) was used for sensing 4-nitrophenol (4NP) [298]. The anodic current of the modified electrode compared to the bare one was ca. 5 times higher (see Figure 25) in acetate buffer at pH 4.5. A linear response was obtained by DPV for 4NP concentration ranging from 0.24 μM to 130.6 μM with a detection limit of 9 nM. In the repeatability test, an RSD value of 2.56% was obtained after six measurements.

**Figure 26:**
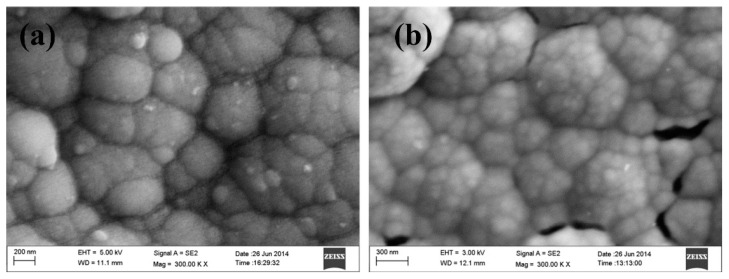
FESEM images of polypyrrole/sodium dodecyl sulphate film, (**a**) before and (**b**) after electrochemical treatment. Modified with permission from [299]. Copyright 2015 Elsevier.

Arulraj et al. fabricated an electrogenerated nano polypyrrole/sodium dodecyl sulphate (ENPPy/SDS) film for the determination of 4NP [299]. Oxidation peak current showed a linear response in the range of 0.1 nM–100 µM with a LOD of 0.1 nM and sensitivity of 4.45 µA µM^−1^. The catalytic effect of ENPPy/SDS film can be explained by the electrochemical treatment in which the initial globular structure (see Figure 26a) is nano cracked and decreases the particle size of the polymer (see Figure 26b). Cracks allowed 4NP diffusion into the polymer matrix through capillary action and act as micro electrochemical cells catalyzing the reaction.

Finally, Table 2 enlists a series of electrochemical sensors based on conducting polymers for the detection of the various emergent aqueous contaminants reviewed in the last five sections. Electrode architecture, name of the conducting polymer, synthetic methodology, analyte, detection technique, LOD, and linear range are provided as well as the reference where the information was taken.

## 4. Conclusions

Electrochemical CP sensors have been developed for versatile and selective detection of different organic and inorganic biologically relevant compounds. The most-used monomers are derivatives of pyrroles, anilines, ethylenedioxythiophenes, and conjugated organic dyes because of their capacity to produce conducting polymers with high conductivity, large surface area, and improvement in electron transfer kinetics, which enhances the electrocatalytic activity of the sensor. Moreover, these conducting polymers can be coupled with many other materials such as graphenes, carbon nanotubes, enzymes, and metal nanoparticles for increasing adsorption of analytes which rises sensitivity and allows biocompatibility. These composites show excellent performance due to the synergetic effects of the conducting polymers with the other components improving the sensitivity, selectivity, and stability of the sensors. Electrochemical polymerization is the most used synthetic methodology for generating the polymeric film and their composites allowing for a precise thickness and morphology modulation. Molecular imprinted polymerization appears as an interesting methodology to obtain high-performance sensors. During the polymerization, the analyte molecule acts as a template that artificially synthesizes receptor structures with specific recognition, highly increasing the sensitivity and selectivity of the sensor. These electrochemical sensors based on conducting polymers display limits of detection that can even reach attomolar concentrations, which satisfy the detection requirements for traces monitoring. The design and fabrication of modified electrodes with highly ordered multilayers remain quite an unexplored research area as well as the development of non-invasive sensors like skin patches or needle-less devices.

## 5. Perspectives

There are still challenges as well as opportunities for the development of novel electrochemical polymeric sensors. Most of the thin polymeric films are generated from bifunctional monomers which leads to the formation of linear or branched polymer structures. By using the right methodology, thin films with high roughness and large surface areas are synthesized showing meso- and macroporosity. Nevertheless, recent efforts in the development of tridimensional rigid polymeric networks with high microporosity have been made resulting in semiconducting thin films that show improved sensitivity and selectivity. In recent years, a series of microporous polymer networks (MPNs) based on multifunctional carbazole monomers on GCE were tested for the analysis of 1,3,5-trinitrobenzene (TNB) [300]. The monomers, which deferred mostly in the number of electroactive carbazole units, were electrodeposited by potentiostatic methods. As the monomers possess multiple carbazole functions around a rigid core unit, the resultant thin film is a three-dimensional network with permanent microporosity. Direct measurement of the specific surface area was determined for the MPNs using krypton gas sorption measurements followed by data analysis with the Brunauer−Emmett−Teller (BET) equation reaching values up to 1297 m^2^g^−1^. The electron-rich MPN surface interacts with electron-poor nitroaromatic analytes via π−π interactions boosting the sensitivity of the electrode, which is closely related to the specific area, showing a current increase of up to 182 between modified and nonmodified GCE. Similar works have also been published where it was demonstrated the importance of producing high microporosity films, starting from multifunctional monomers, for the sensitivity boosting of modified electrodes in the detection of various analytes [301,302,303,304,305].

## Figures and Tables

**Table 1:** Summary of the performance analysis of different electrochemical sensors based on conducting polymers for the aqueous detection of various molecules with a fundamental role in the human body.

Electrode Architecture	Conducting Polymer	Synthesis Method	Analytes	Detection Technique	LOD (µM)	Linear Range (µM)	Ref.
Dopamine							
(PPY)-Ag	Polypyrrole	Self-assembled/liquid phase	DA	LSV	0.00005	0.00005 to 0.003	[93]
AuNP-GP-PEDOT:PSS/GCE	PEDOT:PSS	Self-assembled/liquid phase	DA	DPV	0.0001	0.001 to 300	[119]
Au-PDNs	Polydopamine	Self-assembled/liquid phase	DA, UA, AA, tryptophan	DPV	0.0001	1 to 160	[128]
OPPy/SDS-CNT	Polypyrrole	CA	DA	DPV	0.000136	0.005 to 0.010	[92]
GN/PoP	Poly (o-phenylenediamine)	CV	DA	SWV	0.00016	0.001 to 150	[103]
MWCNTs-COOH/Poly(TB)/GCE)	Poly (toluidine blue)	CV	DA	DPV	0.00039	1 to 300	[127]
ERGO-pEBT/AuNPs	Poly (eriochrome black T)	CV	DA, UA, AA	DPV	0.009	0.5 to 20	[126]
CD-f-PEDOT: PSS	PEDOT:PSS	Spin coating technique	DA, catechol	DPV	0.009596	0.05 to 200	[118]
Poly-β-CD(f-MWCNTs)/PANI	Polyaniline	CV	DA	DPV	0.0164	2 to 24	[99]
PrGO/MnO2	Poly (3,4-ethylenedioxythiophene)	CV	DA, UA, AA	DPV	0.02	0.03 to 45	[116]
MWCNTs/CeO_2_-PEDOT	Poly (3,4-ethylenedioxythiophene)	Self-assembled/liquid phase	DA	DPV	0.03	0.1 to 10	[115]
AuNPs/PANI-co-PoAN/GO	Poly (aniline-co-o-anisidine)	Self-assembled/liquid phase	DA	SWV	0.0334	5 to 100	[101]
pHQ/AuNPs/NF	Poly (hydroquinone)	CV	DA	DPV	0.0419	0.1 to 10	[125]
GCE/PGBHA-afGQDs-MnO_2_	Poly (glyoxal-bis(2-hydro- xyanil))	CV	DA	DPV	0.05	0.1 to 100	[124]
rGo/Pd@PPy NP	Polypyrrole	Self-assembled/liquid phase	DA, UA, AA	DPV	0.056	1000 to 15,000	[89]
POMA-Au	Poly (o-methoxyaniline)	Self-assembled/liquid phase	DA, Folic acid	DPV	0.062	10 to 300	[100]
PEDOT/Au	Poly (3,4-ethylenedioxythiophene)	Self-assembled/liquid phase	DA, UA	DPV	0.07	0.15 to 330	[114]
CuTRZMoO_4_@PPy-n	Polypyrrole	Self-assembled/liquid phase	DA	DPV	0.08	1 to 100	[83]
OPPy/ERGO	Polypyrrole	CV	DA	DPV	0.2	0.4 to 517	[88]
Poly-AHMP	Poly-4-Amino-6-hydroxy-2-mercaptopyrimidine	CV	DA, Acetominphen	DPV	0.2480	2.5 to 25	[104]
PEDOT/LSA	Poly (3,4-ethylenedioxythiophene)	CV	DA	DPV	0.26	0 to 5	[113]
p-ProH/GCE)	Poly (procaterol hydrochloride)	CV	DA, UA	SWV	0.3	1 to 100	[123]
PEDOT/IL/GCE	Poly (3,4-ethylenedioxythiophene)	CV	DA	CV	0.33	0.2 to 328	[117]
PEDOT-LSG	Poly (3,4-ethylenedioxythiophene)	CA	DA	DPV	0.33	1 to 150	[112]
PT/Au/CNT	Polythiophene	Self-assembled/liquid phase	DA	DPV	0.69	1 to 10	[120]
PPy/C#SiO_2_	Polypyrrole	Self-assembled/liquid phase	DA	DPV	0.76	1 to 100	[91]
PPy-SβCD	Polypyrrole	CA	DA	CA	1	Not reported	[87]
Poly phenol red/GCE	Poly phenol red	CV	DA, Acetaminophen	DPV	1.6	20 to 160	[122]
PANI-Au	Polyaniline	Self-assembled/liquid phase	DA	DPV	5.25	7 to 100	[98]
PS/MCPE	Poly (sudan III)	CV	DA	DPV	9.3	10 to 90	[121]
MIPs/ZNTs/FTO glass	Polypyrrole	CV	DA	DPV	Not reported	0.02 to 5	[90]
**Epinephrine**							
EB-Ppy-BSA/GCE	Polypyrrole	Self assemble	EP, tyrosine	SWV	0.0074	0.1 to 400	[129]
(FA)/AuNP/GCE	poly-fuchsine acid	CV	EP, AA, UA	DPV	0.01	0.5 to 792.7	[135]
mpg-C3N4/PANI/CdO	Polyaniline	CA	EP, PR, CFX, mefenamic acid	DPV	0.011	0.05 to 80	[131]
PAPBA(MIPs)/MWCNTs	Poly (3- aminophenylboronic acid	CV	EP	DPV	0.035	0.2 to 800	[133]
Au/ZnO/Ppy/RGO	Polypyrrole	CA	EP, AA, UA	DPV	0.058	0.6 to 500	[130]
MIP/AuNP	Poly (3-Thiophene boronic acid)	CV	EP, tyrosine	DPV	0.076	0.09 to 100	[134]
MWCNT-PANI-TiO2	Polyaniline	Self assemble	EP, tyrosine	DPV	0.16	4.9 to 76.9	[132]
PBCB/graphene/GCE	Poly (brilliant cresyl blue)	CV	EP	CV	0.24	1 to 1000	[136]
**Serotonin**							
p(P3CA)/PGE	Poly (pyrrole-3-carboxylic acid)	CV	SER	AdSDPV	0.0025	0.01 to 1	[137]
AuNPs@rGO/pTBA Pd(C2H4N2S2)2)	Poly 2,2:5,2-terthiophene-3-(p- benzoic acid)	CV	SER, DA	SWV	0.0025	0.02 to 20	[142]
GR/p-AHNSA/SPCs	Poly 4-amino-3-hydroxy1- naphthalenesulfonic acid	CV	SER, DA	SWV	0.003	0.05 to 150	[139]
MWCNTs–CS–poly ( p-ABSA)/GCE	Poly (p-amino benzene sulfonic acid)	CV	SER	DPV	0.08	0.1 to 100	[138]
Fe3O4–MWCNT–poly (BCG	Poly (bromocresol green	CV	SER	DPV	0.08	0.5 to 100	[140]
rGO−PEDOT/PSS	PEDOT:PSS	Self assemble	SER	DPV	0.1	1 to 10	[141]
**Uric Acid**							
Ox-PEDOT-nf/PGE	Poly (3,4 ethylenedioxythiophene)	CV	UA	DPV	0.0013	0.01 to 20	[147]
MIP/RGO	2-amino-5-mercapto-1, 3, 4-thiadiazole	CV	UA and tyrosine	DPV	0.0032	0.01 to 100	[149]
PSA/ERCG/GCE	Poly (sulfosalicylic acid)	CV	UA and isoniazid	DPV	0.012	0.02 to 15	[150]
α-Fe_2_O_3_/PAn NTs	polyaniline	Self-assembled/liquid phase	UA	DPV	0.038	0.01 to 5	[145]
6-TG/GCE	6-thioguanine	CV	DA, UA, XA and HXA	DPV	0.06	2 to 1600	[152]
AuNPs/poly-TrB/GCE	Au-nanoparticles/poly-Trypan Blue	CV	UA, cysteine and tyrosine	DPV	0.07	1 to 550	[151]
POMANS-MWCNT/GPE	Polyortho-methoxyaniline	Self-assembled/liquid phase	UA and folic acid	LSV	0.157	0.6 to 52	[146]
PEDOT/GO/ITO	Poly (3,4 ethylenedioxythiophene)	Self-assembled/liquid phase	UA	DPV	0.75	2 to 1000	[148]
p-TPP/PPy/GO	polypyrrole	Self-assembled/liquid phase	UA	DPV	1.15	5 to 200	[144]
**Ascorbic Acid**							
Graphite/PAMAN-CNT/p( Neutral red)	Neutral Red	CV	AA	CA	0.053	0.2 -2500	[156]
GCE/NiNP/CNT/PANI	PANI	CV	AA	DPV	0.1	1.0 to 450	[154]
GCE/PGBHA	Poly (glyoxal-bis(2-hydroxyanil))	CV	AA	DPV	0.26	1 to 8	[159]
GCE/PPy@Celluloce	PPy	Homogeneous synthesis	AA	DPV	0.75	10 to 50	[153]
GCE/CNT-CA/PEDOT	PEDOT	CA	AA	CA	4.2	0.1 to 20 mM	[158]
CPE/polyamic acid-AuNP	Polyamic acid derivates	Homogeneous synthesis	AA	CA	18.5	10 to 1000 mM	[155]
GCE/PEDOT	PEDOT	CV	AA	CV	45	30 to 500 mM	[157]
**Glucose**							
GC/PEDOT-CNT-Cu2ONP	PEDOT	Homogeneous synthesis	Glucose	CA	0.040	0.495 to 374 mM	[187]
GC/PEDOT-GO/CuNP	PEDOT	CV	Glucose	CA	0.047	0.1 to 1300	[186]
Pt/PANI-MMT/GS-GOx	PANI	CV	Glucose	CA	0.1	10 to 1940	[167]
Au/MWCNT/Pdplate/ GOx-PAB-PdNP/CS	Poly (3-anileneboronic acid)	CV, CA	Glucose	CA	0.1	2 to 4500	[168]
GC/PANI-NiONP	PANI	Homogeneus preparation	Glucose	CA	0.19	Up to 100	[179]
SPCE/AuNP/pTBA-MICP	Poly (tertiophene),	CV	Glucose	Potentiometric	0.19	0.32 to 1000	[185]
GC/pPD/CuNP	Poly (o-phenylenediamine)	CV	Glucose	Ca	0.25	5.0 to 1600	[179]
Graphite/pPy/Ni(OH)2NP	pPy	CA	Glucose	CA	0.3	1 to 4860 mM	[175]
FTO/AnB/AuNP	Poly (aniline blue)	CV	Glucose	CA	0.4	0 to 50	[178]
GC/PEDOT.PSS/NiNP	PEDOT-PSS	controlled potential coulometry	Glucose	CA	0.69	2.5 to 1115	[184]
GC/PEDOT-GO/NiNP	PEDOT	CV	Glucose	CA	0.8	1 to 5100	[183]
Pt/PANI-GRA/GS-GOx	PANI	CV	Glucose	CA	2.8	10 to 1480	[166]
GC/pPy-AgNP	pPy	Homogeneos polymerization	Glucose	CA	3.6	25 to 2500	[174]
GE/CNT-COOH-pAT/AuNP	Poly (2-aminothiophenol)	chemical polymerization	Glucose	LSV	3.7	100 to 30,000	[177]
PGE/PAMAN/pMB-GHD	Poly (methylene blue)	CV	Glucose	flow injection analysis	4.0	0.001 to 1.0	[173]
GC/pEDOT-PBA	Poly (EDOT-PBA)	CV	Glucose	EIS	5.0	100 to 50,000	[182]
GC/PANI-PVP-AuNP/ GOx-Nafion	PANI-PVP	CV	Glucose	CA	10.0	0.05 to 2.25	[165]
ITO/pPD/AgNP	Poly (o-phenylenediamine)	CV	Glucose	CA	12.0	150 to 13,000	[188]
ITO/PP3C-GO/GOx	Poly (pyrrole-3-carboxilic acid)	CV	Glucose	CA	50.0	1 to mM	[163]
GC/PANI/AuNP	PANI	Drop-cast of solution polymer	Glucose	EIS	100.0	300 to 1000	[176]
G/p(EDOT-PdBPI)n-(HKCN)m-GOx	PEDOT	CV	Glucose	CA	180.0	250 to 2500	[170]
PGE/PEDOT.PSS-CuONP	PEDOT.PSS	Homogeneous synthesis and drop-cast	Glucose	CA	230.0	Up to 10 mM	[181]
Pt/PEDOT-PAA-GOx	PEDOT	CA	Glucose	CA	290.0	960 to 3000	[171]
Pt/PEDOT-BSA/AuNP-GOx	PEDOT	CV	Glucose	CV, LSV, CA	Not reported	0.416 to 50 mM	[169]
Pt/PANI/GOx/PU/E-PU	PANI	CA	Glucose	CA	Not reported	0–20 mM	[164]
GC/PHMeDOT	Poly (hydroxymethyl-3,4-ethylendioxythiophene)	CA	Glucose	CA	Not reported d	1–9 mM	[180]
**Hydrogen Peroxide**							
Nafion/HRP/ATh-γ-PGA/GE	ATh-γ-PGA	CV	H_2_O_2_	DPV	0.0000030	0.00001–0.010	[197]
HRP/AuNPs/rGO/PEDOT:PSS/SPGE	PEDOT:PSS	CV	H_2_O_2_	CA	0.08	5–400	[196]
Ag/PMB/GS/GCE	PMB	CV	H_2_O_2_	CA	0.15	0.5–1112	[204]
PEDOT/PBNPs/GCE	PEDOT	CA	H_2_O_2_	CA	0.16	0.5–839	[201]
PEDOT/PBNPs/Pt	PEDOT	SV	H_2_O_2_	CA	1.4	5–1000	[202]
PPy3C-PPy/MPrPt/BDD	PPy3C-PPy	CV	H_2_O_2_	CA	2.0	5–49,000	[205]
SBP/poly (EGDE-AA-ANI)/GCE	PANI	radical polymerization	H_2_O_2_	CA	2.2	5.0–50	[199]
HRP/PAN-PNMThH	PAN-PNMThH	CV	H_2_O_2_	CA	3.2	5–60,000	[198]
poly2AB/AuNPs/PGE	poly2AB	CV	H_2_O_2_	CA	36.7	60–100,000	[203]

**Table 2:** Summary of the performance analysis of different electrochemical sensors based on conducting polymers for the detection of various water emergent pollutants.

Electrode Architecture	Conducting Polymer	Synthesis Method	Analytes	Detection Technique	LOD (µM)	Linear Range (µM)	Ref.
Diverse Pharmaceuticals							
Poly [(3, 6-diamino-9-ethylcarbazole)]/GCE	Poly [(3, 6-diamino-9-ethylcarbazole)]	CV	E2	EIS	0.36 aM	1 aM to 10 μM	[223]
MnO2-Sb2O3/PANI//FTO	PANI	CV	ASA	DPV	0.0002	0.0012–0.22868	[241]
PPy/sol-gel/SiO2@AuNPs MIP/Au electrode	PPy	CV	ASA	SWV	0.0002	0.001–0.01	[242]
PPy/PB/GCE	PPy	CV	PR	DPV	0.00053	0.001–100	[228]
3D-HPG/PTH/GCE	PTH	CV	MNZ	DPV	0.001	0.05–70	[209]
rGO/PPR/GCE	PPR	CV	CFX	DPV	0.002	0.002–0.05	[219]
Cu2+-PANI-Nano-ZSM-5/GCE	PANI	CV	PR	DPV	0.008	0.015–800	[237]
CS-MWCNTs+TiO2 NPs/PCC/nanoporous GCE	PCC	CV	Acyclovir	DPASV	0.01	0.03–1	[214]
PEBT/GCE	PEBT	CV	Acyclovir	DPV	0.012	0.03–0.3	[213]
P3MT/RGO/GCE	P3MT	CV	PR	DPV	0.025	0.2–2.5	[232]
PLum/f-MWCNTs/GCE	PLum	CV	PR	DPV	0.025	0.04–32.2	[233]
PEDOT:PSSLi/GCE	PEDOT	CA	PR	DPV	0.05	0.14–400	[234]
PEDOT:PSSLi:MWCNT/GCE	PEDOT	CA	PR	AdSDPV	0.08	1.5–500	[234]
PNB/GCE	PNB	CV	PR	DPV	0.08	0.2–16.2	[231]
DMIP/CPE	Poly(AMTEOS)	CV	MNZ	DPV	0.091	0.4–200	[207]
HRP/Pol/Pt	Pol	CA	E2	DPV	0.105	0.1–200	[222]
MIP/AuNPs/GCE	PME	CV	MNZ	DPV	0.12	0.5–1000	[208]
SDS/PEB/CPE	PEB	CV	CFX	DPV	0.183	50–90	[218]
Poly(naphthol green B)/CPE	Poly(naphthol green B)	CV	PR	CV	1.6	20–70	[230]
Poly(rhodamine B)/CPE	Poly(rhodamine B)	CV	PR	CV	2.2	20–90	[229]
MWCNT/PANI/AuE	PANI	CV	PR	CA	2.9	0.5–630	[235]
**Hydrazine**							
GCE/PDA@GO	Poly dopmanie	Homogeneous polymerization	Hidrazine	SWV	0.01	0.03 to 100	[262]
SPE/PDDA@[Cu(CN6)]	Poly (diallydimethylamonium chloride)	Commercial polymer	Hidrazine	CA	0.01	0.03 to 570	[257]
GCE/3D-PEDOT/CuxO	PEDOT	CA	Hidrazine	CA	0.2	0.5 to 600	[260]
GCE/PEDOT/ZnO	PEDOT	Chemical polymerization	Hidrazine	CA	0.207	0.5 to 48	[259]
GCE/PALS	Alizarin red S	CV	Hidrazine	CA	0.28	1 to 600	[261]
GCE/PPy-LS	Ppy	Galvanostatic	Hidrazine	CA	1.6	1–80	[253]
Paper/PEDOT/ZnO/Nf	PEDOT PSS	commercial polymer	Hidrazine	CA	5.0	10 to 500	[258]
FTO/PANI-gC3N4/AgNP	PANI	CV	Hidrazine	CV	300.0	5–300 mM	[254]
CPE/PRA@NiFe2O4NP	Poly (rhodamine)	Chemical oxidation with KMnO4	Hidrazine	CA, CV	Not reported	1–50 mM	[256]
GCE/pyrolized PANI/CuNP/Nf	PANI	Chemical polymerization	Hidrazine	CV	Not reported	10 to 100 mM	[255]
**Nitrites**							
AuNP/ PEDOT/PMo_9_V_3_/PEI/GCE	PEDOT	CV	nitrite	CA	0.001	0.0025–1430	[271]
CoNS/GO/PPy/GCE	PPy	CV	nitrite	CA	0.015	1.0–3200	[273]
PdNPs/poly (1,5-DAN)/MWCNTs/GCE	Poly (1,5 DAN)	CV	nitrite	CA	0.08	0.25–100	[272]
nHAp/PEDOT/GCE	PEDOT	CA	nitrite	CA	0.083	0.25–1050	[269]
CQDs/PEDOT/GCE	PEDOT	CA	nitrite	CA	0.088	0.5–1110	[268]
Au/PEDOT-SH/PEDOT/GCE	PEDOT-SH/PEDOT	CV	nitrite	CA	51.0	150–1000	[270]
**Phenolic Compounds**							
*f*-SWCNTs/PEDOTM/GCE	PEDOTM	CA	catechin	CV	0.013	0.039–40.84	[284]
Poly (NG B)/CPE	Poly (NG B)	CV	HQ	DPV	0.01	0.1–110	[286]
			CC	CV	0.19	0.20–90	
			HQ	CV	0.20	0.20–90	
Cu-PPy/GCE	PPy	CA	CC	CA	0.010	0.05–1000	[287]
				DPV	1.17	10–1750	
1D PEDOT-Gr/Ta	PEDOT	CV	HQ	DPV	0.06	5–250	[285]
			CC	DPV	0.08	0.4–350	
			RC	DPV	0.16	6–2000	
**Nitroaromatic Compounds**							
ENPPy/SDS/GCE	ENPPy	CV	*p*-NP	SWV	0.0001	0.0001–100	[299]
Poly(35DT)/GE	Poly(35DT)	CV	4-NP	DPV	0.09	0.24–130.6	[298]
Poly (p-ABSA)/GrE	Poly (p-ABSA)	CV	o-NP	SDV	0.28	0.3–800	[296]
			p-NP	SDV	0.3	0.3–700	
			m-NP	SDV	0.5	0.3–700	
PAR/GCE	PAR	CV	NF	DPV	0.33	3.0–50	[295]
			NIT	DPV	0.73	10.0–40	
			FL	DPV	1.56	50–140	
PME/MWCNT*/SPCE	PME	CV	NFZ	DPV	0.006	0.05–2.0	[297]
			FZD	DPV	0.007	0.05–2.0	
			NFT	DPV	0.012	0.05–2.0	
			FTD	DPV	0.014	0.05–5.0	
AuNp/P(o-PDA-co-ANI)/GCE)	P(o-PDA-co-ANI)	CV	DNT	CV	7.03	11–220	[294]
			TNT	CV	9.25	11–176	
			Tetryl	CV	13.23	14–348	

## Data Availability

No new data were created or analyzed in this study. Data sharing is not applicable to this article.

## Abbreviations

1:5-DAN	1,5-Diaminonaphthalene
35DT	3,5-Diamino-1,2,4-Triazole
3-TBA	3-Thiophene boronic acid
AA	Ascorbic acid
AcA	Acrylic Acid
AdSDPV	Adsorptive differential pulse stripping voltammetry
afGQDs	Amino-functionalized graphene quantum dots
AFM	Atomic force microscopy
AGCE	Anodized glassy carbon electrode
AgNCs	Silver nanocrystals
AHMP	Poly-4-Amino-6-hydroxy-2-mercaptopyrimidine
AMTEOS	anilinomethyltriethoxysilane
APS	Ammonium persulphate
APTMS	3-aminopropyltriethoxysilane
ASA	Acetylsalicylic Acid
Ath	3-Aminothiophene
AuNPs	Gold Nanoparticles
BDD	Boron-Doped Diamond
BSA	Bovine serum albumin
C#	Carbon-coated mesoporous
CA	Chronoamperometry
CC	Catechol
CD	Cyclic dextrin
CE	Counter electrode
CFX	Ciprofloxacin
CNT	Carbon nanotubes
CoNS	Cobalt Nanostructures
CPs	Conducting polymers
CPE	Carbon paste electrode
CQDs	Carbon Quantum Dots
CS	Chitosan
CV	Cyclic voltammetry
DA	Dopamine
DMF	N,N-Dimethylmethanamide
DNT	2,4-Dinitrotoluene
DPASV	Differential Pulse Anodic Striping Voltammetry
DPV	Differential pulse voltammetry
E	Voltage
E2	17-β-Estradiol
EB	Electron Beam
EBT	Eriochrome black T
EDOT	(3,4-ethylenedioxythiophene)
EGDE	Ethylene Glycol Diglycidyl Ether
ENPPy	Nano Poly (Pyrrole)
EP	Epinephrine
ERGO	Electrochemical reduced graphene oxide
FA	Poly-fuchsine acid
FESEM	Field emission scanning electron microscopy
f-MWCNTs	Functionalized multi-walled carbon nanotubes
f-SWCNTs	Functionalized Single-Walled Carbon Nanotubes
FTD	Furaltadone
FTIR	Fourier-transformed infrared spectroscopy
FTO	Fluorine Tin Oxide
FZD	Furazolidone
GCE	Glassy Carbon Electrode
GO	Graphene oxide
GOx	Glucose Oxidase
GP	Graphene
GS	graphene carbon spheres
HQ	Hydroquinone
HRP	Horseradish Peroxidase
HXA	Hypoxanthine
i	Current
IL	Ionic liquid
ITO	Indium thin oxide
IUPAC	International Union of Pure and Applied chemistry
LOD	Limit of detection
LSG	Laser scribed graphene
LSV	Linear swipe voltammetry
MIP	Molecular Imprinted Polymer
MNZ	Metronidazole
MOF	Metal- organic framework
MPrPt	Mesoporous Platinum
MS	Mass spectroscopy
MWCNT	Multi Wallet carbon nanotubes
Nf	Nano fiber
NFT	Nitrofurantoin
NFZ	Nitrofurazone
NG B	Naphthol Green B
nHAp	Nano-sized Hydroxyapatite
NP	Nitrophenol
NPs	Nanoparticles
OPPy	Overoxidized electropolymerized polypyrrole
p(P3CA)	Poly (pyrrole-3-carboxylic acid)
P3-TBA	Poly 3-Thiophene boronic acid
P6-TG	Poly(6-thioguanine)
p-ABSA	p-Aminobenzene Sulfonic Acid
p-AHNSA	Poly 4-amino-3-hydroxy-1-naphthalenesulfonic acid
PAMT	Poly (2-amino-5-mercapto-1, 3, 4-thiadiazole)
PANI	Polyaniline
PANI-co-PoAN	Poly (aniline-co-o-anisidine)
PAPBA	Poly (3-aminophenylboronic acid)
PAR	Poly (Alizarin Red)
PBCB	Poly (brilliant cresyl blue)
PBS	Phosphate buffer solution
PCC	Poly-catechol
PDA	Phenylenediamine
PdNPs	Palladium Nanoparticles
PDNs	Polydopamine nanospheres
PEB	Poly (Evans Blue)
pEBT	Poly (eriochrome black T)
PEDOT	Poly (3,4-ethylenedioxythiophene)
PEDOT:PSS	Poly (3,4-Ethylenedioxythiophene):Polystyrene Sulfonate
PEDOTM	Poly (Hydroxymethylated-3,4-Ethylenedioxythiophene)
PEDOT-SH	Poly (Thiomethyl 3,4-Ethylenedioxythiophene)
PGBHA	Poly (glyoxal-bis(2-hydro- xyanil)
PGE	Pencil Graphite Electrode
pHQ	Poly (hydroquinone)
PMB	Poly (Methylene Blue)
PME	Poly (Melamine)
PNEDA	Poly (N-(Naphthyl) ethylenediamine dihydrochloride) nanofibers
Pol	4,7-bis(5-(3,4- Ethylenedioxythiophene)thiophen-2-yl)Benzothiadiazole
poly 2AB	Poly (2-aminophenylbenzimidazole)
Poly(BCG)	Poly (bromocresol green)
poly(p-ABSA)	Poly (p-amino benzene sulfonic acid)
Poly(TB)	Polytoluidine blue
poly-TrB	Poly-Trypan Blue
POMA	Poly (o-methoxyaniline)
PPR	Poly (Phenol Red)
p-ProH	Poly (procaterol hydrochloride)
PPy	Polypirrrole
PPy3C	Poly (Pyrrole-3-Carboxylic Acid)
PR	Paracetamol
PS	Polysudan III
PSA	Poly (sulfosalicylic acid)
PTH	Polythionine
p-TPP	Polytetraphenylporphyrin
pTSA	p-toluene sulphonic acids
PVP	Polyvinylpyrrolidone
RSD	Relative standard deviation
RC	Resorcinol
RE	Reference electrode
rGO	Reduced Graphene Oxide
SBP	Soybean Seed Coat Peroxidase
SDS	Sodium Dodecyl Sulphate
SEM	Scanning electron microscopy
SER	Serotonin
SPCs	Screen printed carbon sensor
SWV	Square Wave Voltammetry
SβCD	Sulfonated β-cyclodextrin
TBA-TFB	tetrabutylammonium tetra- fluoroborate
TEM	Transmission electron microscopy
TEOS	tetraethyl orthosilicate
Tetryl	2,4,6-Trinitrophenylmethylnitramine
TNT	2,4,6-Trinitrotoluene
UA	Uric acid
WAXD	Wide angle X-Ray diffraction
WE	Working electrode
XA	Xanthine
ZNRs	Zinc Nano rods
ZNTs	Zinc nanotubes
γ-PGA	Poly (γ-Glutamic Acid)
